# Multi-omics comprehensive analysis identified KIF22 and KRAS as highly synthetic lethal pairs for triple-negative breast cancer

**DOI:** 10.3389/fonc.2026.1748954

**Published:** 2026-02-06

**Authors:** Shichen Miao, Xiao Wang, Qiming Gu, Chengyu Bian, Rui Fan, Qichao Ni, Yi Wang, Zhigang Zhuang

**Affiliations:** 1 Department of Breast Surgery, Shanghai Key Laboratory of Maternal Fetal Medicine, Shanghai Institute of Maternal-Fetal Medicine and Gynecologic Oncology, Shanghai First Maternity and Infant Hospital, School of Medicine, Tongji University, Shanghai, China; 2 Department of Thyroid and Breast Surgery, Affiliated Hospital of Nantong University, Medical School of Nantong University, Nantong, China; 3 School of Public Health, Nantong University, Nantong, Jiangsu, China; 4 Institute for Health Development, Nantong University, Nantong, Jiangsu, China; 5 Department of Thoracic Surgery, Shanghai Pulmonary Hospital, School of Medicine, Tongji University, Shanghai, China; 6 School of Pharmacy, Nantong University, Nantong, China; 7 Department of Hepatobiliary Surgery and Liver Transplantation, Liver Cancer Institute, Zhongshan Hospital, Fudan University, Shanghai, China

**Keywords:** machine learning, multi-omics, synthetic lethality, triple-negative breast cancer, tumor heterogeneity

## Abstract

**Background:**

Triple-negative breast cancer (TNBC) is an aggressive subtype of breast cancer with a poor prognosis and limited treatment options. Synthetic lethality (SL) represents a significant therapeutic strategy that selectively kills cancer cells without affecting normal cells by targeting the synergistic interaction of two genes. The SL strategy offers new avenues for targeted therapy in TNBC. Although challenges remain—such as drug resistance and biomarker selection—advancing research in SL activity holds promise for delivering clinical benefits to patients.

**Methods:**

Multi-omics data, including single-cell RNA sequencing (scRNA-seq), spatial transcriptomics (ST), and bulk RNA sequencing (bulkRNA-seq), were utilized to characterize TNBC heterogeneity and identify genes driving SL activity. Additionally, CytoTRACE analysis assessed tumor differentiation potential in high SL (HSL) activity cell subpopulations, Slingshot reconstructed pseudo-temporal trajectories, and CellChat constructed intercellular communication networks to evaluate interactions among TNBC microenvironment cell subpopulations. Combining high-dimensional weighted gene co-expression network analysis (hdWGCNA) with machine learning, key regulatory genes associated with the HSL cell phenotype were identified. Finally, a benchmarking framework was employed to select the most predictive algorithmic model, with feature contributions evaluated via SHapley Additive Explanations (SHAP) analysis. The identified genes were analyzed *in vivo* and *in vitro* through molecular biology experiments and animal experiments.

**Results:**

A novel HSL subtype of TNBC malignant cells has been identified, exhibiting enhanced stem cell-like properties, stronger intercellular communication capabilities, and involvement in more tumor-associated signaling pathways. Ten characteristic genes identified through five machine learning (PGP, KIF22, CCNB1, RPA3, BCL2L12, SMC2, MKI67, PBK, CDK1, and MIS18A) are significantly upregulated in TNBC malignant cells, and their high expression correlates with poor prognosis in TNBC patients. Benchmarking validated the superior performance of the random forest algorithm. Finally, through experimental verification, it was concluded that KIF22 and KRAS are synthetic lethal pairs for TNBC.

**Conclusion:**

In conclusion, this study systematically characterized the heterogeneity of TNBC and explored the association between SL activity and disease progression through a comprehensive analysis of the interactions between SL pairs and malignant TNBC cells. Our findings contribute to a deeper understanding of the molecular mechanisms underlying TNBC initiation and development. Based on bioinformatics analyses, we experimentally validated KIF22 and KRAS as a synthetic lethal gene pair in TNBC. Functional experiments demonstrated that the knockdown of KIF22 in KRAS-mutated TNBC cells or the knockdown of KRAS in TNBC cells with low expression of KIF22 gene significantly inhibited cell proliferation. Given the high prevalence of KRAS mutations in TNBC, KIF22 represents a promising therapeutic target for synthetic lethal intervention. Furthermore, *in vivo* xenograft models confirmed that concurrent knockdown of murine KIF22 and KRAS effectively inhibited tumor progression. Collectively, these results establish KIF22 and KRAS as a TNBC-specific synthetic lethal pair with strong potential for guiding future SL-based drug discovery efforts.

## Graphical Abstract

**Figure:**
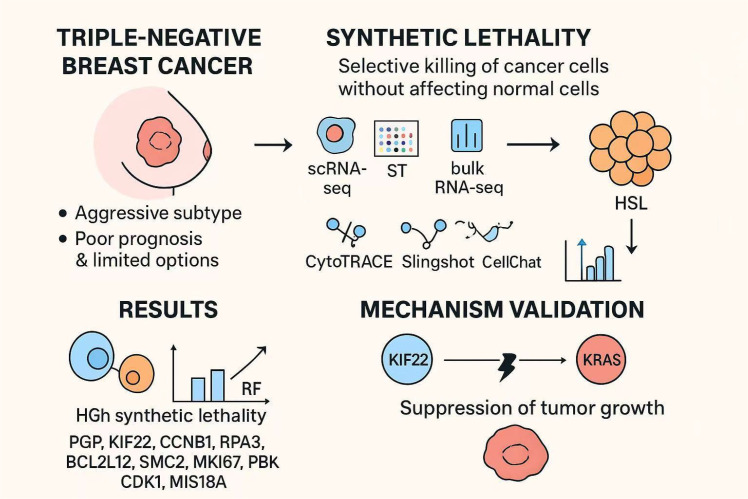

## Introduction

Triple-negative breast cancer which makes up 15% to 25% of all occurrences of breast cancer, is an aggressive subtype of the disease with a high recurrence rate and high risk of metastasis (1). It is most commonly found in premenopausal women, with typical characteristics including cellular expression of progesterone and estrogen receptors (*PR*, *ER*) of ≤1%, human epidermal growth factor receptor 2 (*HER2*) expression between 0 and 1+, and a 5-year survival rate of approximately 11% in patients with advanced tumors under limited treatment options (2, 3). Lifestyle, endocrine function, and genetics are tightly linked to the complicated etiology of TNBC. A number of signaling pathways that promote tumor growth, invasion, and metastasis are dysregulated in it, including the *PI3K/AKT/mTOR*, *MAPK/ERK*, and *JAK/STAT* pathways (4). In recent years, immunotherapy and novel targeted drugs have demonstrated certain efficacy in the treatment of TNBC (5), and hence, further research into the immune microenvironment and biomarkers of TNBC is expected to improve prognosis and treatment outcomes.

In 1946, Dobzhansky established the concept of synthetic lethality (SL), which describes the occurrence of cell death brought on by the simultaneous inactivation of two non-lethal genes (6). By focusing on the distinct genetic flaws or metabolic anomalies of tumor cells, this approach may target the vulnerability of tumor cells and destroy them specifically, marking a significant advancement in precision cancer therapy. Researchers have been able to more thoroughly examine artificially lethal interactions in tumor cells in recent years owing to the advancement of gene editing tools like CRISPR-Cas9 and the improvement of high-throughput screening techniques. Chan et al. conducted a comprehensive CRISPR-Cas9 knockout screening in uveal melanoma and found that disruption of the *CDS1/CDS2* axis leads to the accumulation of precursor phosphatidic acid and lipid droplet and a decrease in phosphoinositides phosphatidylinositol and phosphatidylinositol monophosphate, demonstrating a SL interaction between *CDS1* and *CDS2* (7). Likewise, Pruett et al. identified a new SL interaction in pleural mesothelioma based on microRNA-497-5p screening through *PKMYT1* and *WEE1* (8). Furthermore, Feng et al. found that *DOCK1* levels determine the antitumor activity of metformin, and the efficacy of metformin depends on *DOCK1* levels. Combining metformin with *DOCK1* inhibition may provide a personalized treatment strategy based on SL for hepatocellular carcinoma patients with metformin resistance (9).

In TNBC, numerous studies have also demonstrated the enormous potential of SL in tumor treatment. *BRCA1/2* gene mutations are common genetic defects in TNBC. When *BRCA* genes are mutated, cells rely on other DNA repair pathways to repair DNA damage. *PARP* (polyADP-ribose polymerase) plays an important role in DNA repair. When *BRCA* genes are mutated, inhibiting *PARP* can block DNA repair, leading to the accumulation of DNA damage and ultimately causing cell death (10). Tsoi et al. revealed that Ivabradine induces *BRCA*ness by degrading *RAD51* through the *ATF6-FBXO24* axis, thereby enhancing the efficacy of *PARP* inhibitors in non-germline *BRCA*-mutated TNBC (11). Additionally, study has shown that metabolic shifts (such as changes in *PKM2* and tumor glycolysis) may regulate the lineage plasticity of TNBC and induce SL (12). Zhang et al. found that the *Aurora-A/ERK1/2/mTOR* axis promotes the progression of TNBC tumors, and dual targeting of *Aurora-A/mTOR* shows SL (13). The aforementioned study indicates that SL is a significant biological phenomenon that offers novel, focused cancer therapy approaches. More accurate and efficient cancer treatment techniques can be created by figuring out and using SL mechanisms.

In our study, we utilized multi-omics data, including single-cell RNA sequencing (scRNA-seq) and spatial transcriptomics (ST) technology, to comprehensively analyze the tumor microenvironment of TNBC patients. We detailed the distribution characteristics and expression features of cell subpopulations and SL activity and revealed the heterogeneity and biological functional alterations among malignant cancer cells and within cells. By combining the high-dimensional weighted gene co-expression network analysis (hdWGCNA) with machine learning algorithms, we identified hub biomarkers determining SL activity in TNBC, providing a stratified strategy for predicting patients who may benefit from targeted SL therapy. We further employed SHapley Additive exPlanations (SHAP) for interpretability analysis to quantify the specific contribution of each feature to the model’s predictive outcomes, thereby aiding in the exploration of new SL targets, combination therapy strategies, and biomarker development to improve treatment outcomes and patient survival rates in TNBC patients.

## Methods

### Data collecting and analyzing

All scRNA-seq data were obtained from the GEO database, including GSE161529, GSE176078, and GSE246613, which collectively included 67 TNBC samples. Bulk-RNA sequencing data from 604 TNBC and 113 normal samples were collected from the GEO (GSE58812, GSE31519), TCGA (TCGA-TNBC), and cBioPortal (ID=brca_metabric) databases separately. The ST data was obtained from the study by Sunny Z Wu (14). **Supplementary Table S1** contains comprehensive details on all included datasets.

For scRNA-seq data, we retained cells with mitochondrial read counts not exceeding 20% or with more than 200 detected genes and excluded genes expressed in fewer than three cells and with expression levels less than 200 or exceeding 7,000 cells. 245,924 high-quality cells in total were kept for further analysis after rigorous quality control and Seurat standardization. The “FindVariableFeatures” function was used to identify the top 3,000 highly variable genes. Principal component analysis (PCA) was then used to reduce the dimensionality of the single-cell RNA sequencing data. To more clearly show the differences between samples, the “Harmony” R package was used to correct for batch effects. Using the “FindClusters” function, cell clustering was carried out using a resolution setting of 0.8. The primary emphasis of cell subpopulation annotation is on highly expressed genes with distinctive expression patterns and well-known classic cell markers.

For ST data, spots with extremely low total UMI counts or excessively high mitochondrial gene content were excluded to ensure data quality. Preprocessing and spatial segmentation of distinct tissue regions were performed using Seurat and SCTransform for normalization and unsupervised clustering. The SPOTlight algorithm was employed for cell type annotation, while cell subpopulations were annotated based on H&E staining combined with cluster-specific marker genes. “SpatialDimPlot” and “SpatialFeaturePlot” were employed to visualize spatial transcriptomic maps, enabling in-depth characterization of tissue architecture and its distributional features (15, 16).

### SL activity gene set scoring

175 TNBC-related SL genes were obtained from the SynLethDB database under the catalog of disease types (https://synlethdb.sist.shanghaitech.edu.cn/) (**Supplementary Table S2**). To quantify the expression levels of SL activity at the single-cell scale, five algorithms—AUCell, UCell, singscore, ssGSEA, and AddModuleScore—were developed to assess gene set enrichment and activity in scRNA-seq data (17–21). UCell evaluates enrichment through the application of normalized rank-based scores, providing a standardized metric for gene set activity. In contrast, AUCell integrates the ranked order of gene expression values to infer pathway or signature activity, offering a complementary perspective on cellular states. The ssGSEA method produces relative enrichment scores by systematically comparing the expression distribution of genes inside versus outside a predefined set, enabling robust assessment of gene set overexpression. AddModuleScore computes a summary metric by ranking genes, averaging their relative positions, and calculating a weighted average expression value for the specified gene set. This result is subsequently normalized, yielding a final score methodologically aligned with the output of the singscore algorithm. Furthermore, an average activity score can be generated through the row-wise accumulation of normalized feature values across cells or samples, providing an aggregated measure of transcriptional activity for a gene set of interest. Each method offers a unique computational strategy while contributing to the overarching goal of quantifying gene set enrichment in complex biological data.

### Copy number variation analysis

The “inferCNV” R package was employed to distinguish malignant from non-malignant cell subpopulations at the single-cell level in TNBC. This method analyzes scRNA-seq data to examine the relationship between gene expression patterns and their chromosomal locations, detect genomic alterations such as amplifications and deletions, and thereby infer large-scale copy number variations (CNVs) (22). More specifically, inferCNV establishes a CNV inference standard using normal control cells as the reference population. It then compares expression levels between individual cells to identify genomic instability closely associated with tumor cells, thereby distinguishing malignant from non-malignant cells. During data analysis, we employ a normalization strategy to minimize technical noise and accurately detect chromosomal regions exhibiting abnormal expression patterns.

### Intercellular communication analysis

The “CellChat” R package was used to infer ligand-receptor interactions to investigate intercellular communication inside the TNBC microenvironment (23). First, a communication network was built by finding signaling relationships between the identified cell subpopulations. The netVisual_circle function was then used to visualize signal output and input patterns among cell subpopulations. The netVisual_bubble function was then used to investigate individual signaling pathways, emphasizing ligand-receptor interaction axes.

### Pseudotime analysis

The CytoTRACE algorithm is an unsupervised computational framework designed to infer cellular stemness and differentiation potential from single-cell transcriptomic data (24). It generated a unique CytoTRACE score for each cell based on gene expression, with higher scores indicating lower differentiation, greater immaturity, and more pronounced malignant behavior. The ScanoramaCT function was employed to address potential batch effects across different datasets.

### hdWGCNA and differential expression analysis

To elucidate the regulatory mechanisms driving TNBC progression, we combined high-dimensional weighted gene co-expression network analysis (hdWGCNA) with differential expression analysis. By examining module–phenotype correlations, hdWGCNA revealed functionally coherent gene modules strongly linked to malignant traits (25). Genes from these modules were subsequently analyzed to pinpoint central regulatory genes—those with high intramodular connectivity—likely governing malignant transformation and copy number variation-mediated intratumoral heterogeneity in TNBC.

Differential expression analysis identified differentially expressed genes (DEGs, logFC > 0.25) associated with SL activity in malignant tumor cells by comparing the expression profiles of high and low SL activity group (HSL, LSL) cells identified via the FindMarkers function.

### Functional enrichment analysis

Genetic Set Variation Analysis (GSVA) quantified SL activity by evaluating GSVA scores of HSL and LSL gene sets, thereby elucidating changes in biological functional pathways across groups. The Benjamini-Hochberg (BH) method was used for false discovery rate (FDR) correction, with FDR < 0.05 considered statistically significant (26). The “clusterProfiler” R package was employed to conduct Disease Ontology (DO), Kyoto Encyclopedia of Genes and Genomes (KEGG), and Gene Ontology (GO) enrichment analyses to elucidate the potential biological functions associated with the identified key genes. Among these, DO enrichment analysis was utilized to identify disease-associated pathways linked to these genes, and KEGG pathway mapping assessed gene interactions and reaction networks, while GO analysis delved into biological functions across three domains: biological processes (BP), cellular components (CC), and molecular functions (MF) (27).

### Feature gene screening

Spearman correlation analysis was first employed to screen for characteristic genes exhibiting significant positive associations with HSL (P < 0.05). While in the areas of feature selection, model optimization, and bias reduction, machine learning provided significant advantages. Consequently, we employed five machine learning techniques—Random Forest, LASSO, Adaptive BEst Subset Selection (ABESS), Decision Tree, and Gradient Boosting Machine (GBM)—to identify hallmark genes associated with SL activity in TNBC (28–32). Random Forest utilized an ensemble of decision trees to assess feature importance, aiming to pinpoint the most relevant gene candidates. The “glmnet” package was applied to implement LASSO, which used regularization to refine gene selection by removing redundancy. In contrast, GBM iteratively corrected errors made by sequential decision trees to improve predictive performance, while Decision Trees partition data recursively to form a hierarchical model. ABESS, a recent advancement in high-dimensional feature selection, was designed to optimize the learning process for sparse models. To ensure the accuracy of our gene selection, we defined the genes that appeared in all five methods as SL core genes.

### Model benchmarking and feature interpretability

Benchmarking refers to comparing different machine learning algorithms on a single task or multiple tasks. The benchmark function provided by the “mlr3” package performed benchmarking by running the resample function on each task to resample and collect results, where eight learners—k-nearest neighbor (KNN), naive Bayes (NB), random forest (Ranger), recursive partitioning and regression trees (RPART), support vector machines (SVM), logistic regression (Log_reg), linear discriminant analysis (LDA), and extreme gradient boosting (XGBoost)—were trained and tested on the same dataset for each task (33). The training set (80%) and testing set (20%) were randomly selected from the dataset. Hyperparameter tweaking was done using five-fold internal cross-validation, and model generalization was evaluated using ten-fold external cross-validation. The average area under the curve (AUC) was used to choose the best prediction model.

SHapley Additive Explanations (SHAP) analysis is a feature-based interpretability method that can be integrated into supervised machine learning models to enhance the credibility of predictions. By identifying interactions between genes and their average contribution to sample predictions, it explains the influence of each feature (gene) on the model’s output (34).

### Cell lines

The following cell lines were cultured under specific conditions: MDA-MB-231, MDA-MB-468, MCF-7, and SUM159 cells in DMEM with 10% fetal bovine serum (FBS) and 1% penicillin-streptomycin; BT474 cells in RPMI 1640 supplemented with 10% FBS and 1% penicillin-streptomycin; Normal breast epithelial cell lines MCF-10A in MCF-10A Cell Complete Medium; Hs 578T cells in Hs 578T Cell Complete Medium; and SK-RB-3 cells in McCoy’s 5A medium with 10% FBS and 1% penicillin-streptomycin. All cells were maintained at 37 °C in a 5% CO2 atmosphere. These cell lines were obtained from Shanghai Zhong Qiao Xin Zhou Biotechnology Co.Ltd. (ZQ0118, ZQ0373, ZQ0071, ZQ1075, ZQ0451, ZQ0080, ZQ0372). FBS, DMEM, RPMI 1640 and McCoy’s 5A were sourced from Gibco(A5670201, 11965092, 11875093, 16600082). Penicillin-streptomycin was purchased from NCM (C100C5), while the MCF-10A and Hs 578T Cell Complete Media were provided by Procell Life Science & Technology (CM-0525, CM-0114).

### Plasmids, lentivirus production and transduction

Prior to transfection, cells were seeded into 6-well plates at a density of 2 × 10^5^ cells per well the preceding day. Subsequently, on the following day, the culture medium was substituted with a serum-free formulation, and then a bispecific antibody was introduced, followed by an 8 hours incubation. Specified lentiviral plasmids, procured from GenePharma, were then introduced into the MDA-MB-231 and SUM159 cells, leveraging Polybrene to facilitate the process. To establish stable expression, the cells were treated with 2.0 μg/mL puromycin (Solarbio) to eliminate those which were not successfully infected, 48 hours post-infection. The shRNA sequences are cataloged in **Supplementary Table S3**.

### Western blot

We first isolated cellular proteins using RIPA buffer (NCM Biotech, WB3100) enriched with protease inhibitors (Epizyme Biomedical Technology, GRF101). These protein extracts were then fractionated on 10% polyacrylamide gels (Epizyme Biomedical Technology) before being electroblotted onto polyvinylidene fluoride (PVDF) membranes. After blocking with 5% nonfat dry milk, the membranes underwent an overnight incubation at 4 °C with primary antibodies specific for *KIF22* (Chengdu Zhengneng Biotechnology Co.Ltd, #220623) and *KRAS* (Proteintech, 12063-1-AP). The membranes were then treated for one hour at ambient temperature with an HRP-linked secondary antibody (Beyotime, A0208). Finally, protein bands were revealed through ECL detection (NCM Biotech, P10300) and documented with a Bio-Rad imaging system, with signal intensities measured using ImageJ software (National Institutes of Health).

### RNA extraction and RT-PCR

We isolated total RNA from the cells using the RNA-Quick Purification Kit (EScience, RN001) according to the manufacturer’s protocol. Subsequently, we synthesized cDNA from 1 μg of total RNA employing HiScript III RT SuperMix specifically designed for qPCR applications. For reverse-transcriptase PCR amplification, we utilized ChamQ Universal SYBR qPCR Master Mix (Vazyme, Q711) on a LightCycler96 instrument (Roche), strictly following the provided guidelines. The specific primer sequences for our target mRNAs can be found in **Supplementary Table S4**, with GAPDH serving as the housekeeping gene and internal control throughout the analysis.

### mIHC staining

The tissue sample was fixed in 4% polyformaldehyde and embedded in paraffin. Tissue sections of 3 μm thickness were then deparaffinized and rehydrated through successive treatments with xylene and graded ethanol, preparing them for multiplex immunofluorescence staining. Following antigen retrieval using microwave heating, incubation with 3% hydrogen peroxide (H_2_O_2_), and a blocking procedure, primary antibody incubation was carried out. The primary antibodies used were panCK (Abcam, ab308262), *KIF22*, and *KRAS*. After incubation with a horseradish peroxidase (HRP)-conjugated universal secondary antibody, immunofluorescent signals were detected using a four-color multi-target immunofluorescence staining kit, including BNKTSA670 and BNKTSA520 (Biosc Biotechnology). The nuclei were counterstained with DAPI. All slides were then imaged using the Pannoramic MIDI II system (3DHISTECH, Hungary).

### Immunohistochemistry

The tissue microarray samples were subjected to deparaffinization and rehydration processes: two rounds of xylene for 5 minutes each, followed by immersion in 100%, 95%, and 70% alcohol for 1 minute each, and then rinsed with distilled water.to expose the antigens, we employed solution #S1699 from Agilent Dako. Following this blocking step, we introduced the primary antibody *KI67* (Abnova, Ab16667) at a dilution of 1:200 and allowed it to incubate overnight in a refrigerated environment at 4 °C. Subsequently, the secondary antibody was applied and left to incubate at room temperature for thirty minutes. Finally, we utilized diaminobenzidine (DAB) as a chromogen to make the target protein visible, thereby completing the immunohistochemical staining process.

### Cell Counting Kit 8 assays

Having calibrated the cell concentration to 1 × 10^4^ cells/mL, 200 μl of this cell mixture was pipetted into each well of a 96-well plate and left to incubate at 37 °C for various intervals—specifically, 0, 24, 48, and 72 hours. Subsequently, each well received 10 μl of the Cell Counting Kit 8 (Biosharp, BS350B) reagent. Following a 2 hours incubation period under the same temperature conditions, the resulting absorbance values were determined at a 450 nm wavelength with a microplate reader (Flash Biotech).

### Flow cytometry

We carried out flow cytometry analysis following established protocols. To examine apoptosis progression and cell cycle status, the cells underwent trypsinization followed by double washing with PBS. For apoptosis evaluation, we utilized an Annexin V-APC-PI apoptosis detection kit (Multi Sciences, AP101), strictly adhering to the manufacturer’s guidelines. We collected 1 × 10^5^ cells, resuspended them in binding buffer, and then treated them with 5 μl of Annexin V and 10 μl of PI, allowing them to incubate for 10–15 minutes at room temperature away from light. The actual flow cytometry measurements were conducted on a CytoFLEX Beckman Coulter platform, with subsequent analysis of cell cycle data performed using Kaluza software.

### Colony formation assay

To assess colony formation, researchers seeded 800 cells per well in a six-well plate and let the cultures ride in an incubator for roughly ten days. Any cluster boasting more than fifty cells was deemed a clone, at which point the colonies were stained with crystal violet, captured on camera, and counted up.

### Wound healing assay

For wound healing assays, cells were seeded in six-well plates and incubated for 24 hours. A scratch was made using a 10 μL pipette tip, followed by washing with PBS. Images were captured at 0h, 24h, and 48h using an Olympus Imaging System Microscope. Migration was assessed using ImageJ and analyzed with Prism 9 software.

### Transwell cell assay

The migration and invasion capabilities of the cells were assessed utilizing Transwell inserts (Corning, featuring 8 µm pore diameters), which were employed either in their native state or following a Matrigel coating. A cell suspension of 1 × 10^5^ cells in 100 µL of serum-free culture medium was introduced into the upper compartment, whereas the lower reservoir was supplemented with 600 mL of culture medium enriched with 30% fetal bovine serum. Following a 24 hours incubation period, the membranes underwent fixation with methanol and subsequent staining with crystal violet. Migrated cells present in the lower chamber were quantified through microscopic examination, with cell counts per visual field meticulously documented for analysis.

### Animal experiments

We established a research protocol beforehand and formal registration was completed. The animal experiments were conducted under project license #S20251011-004, which received the green light from Nantong University’s Animal Ethics Committee and fell in line with the university’s animal care and use policies. We procured female BALB/c nude mice weighing between 18–20 grams from the Animal Laboratory Center at Nantong University. Transfected MDA-MB-231 cells (8 × 10^6^) were gathered and mixed with a solution containing both culture medium and Matrigel. This blend was then injected just beneath the skin of 18 nude mice. Two months down the road, the mice were humanely euthanized, and tumors were surgically removed and weighed to assess their development. When treatment began, the mice with tumors were randomly sorted into different groups. Every three days, tumor dimensions were gauged with calipers, and tumor volume was determined by applying the formula: Tumor volume (mm³) = length (mm) × width (mm) × depth (mm) × 0.52.

### Statistical analysis

Leveraging R (version 4.1.3), we subjected all data to rigorous analysis and visualization. To compare outcomes between two groups, we employed either the Mann-Whitney U test or Student’s t-test, depending on whether the data satisfied the underlying assumptions for each test. For categorical variables, we utilized chi-square tests, while the two-sided log-rank test helped us compare Kaplan-Meier survival curves. To explore relationships among continuous data, we turned to Pearson’s correlation analysis. Unless otherwise specified, all R packages operated with their factory default settings. Statistical significance was determined using a two-tailed test, with the conventional threshold set at P < 0.05.

## Results

### Single-cell microenvironment atlas of TNBC

A total of 67 samples after integrating three single-cell datasets were included in this study. The distributions of nFeature_RNA, nCount_RNA, percent.MT, percent.HB, and percent.Ribosome were discovered by quality control analysis. Each cell’s ribosome was used to evaluate the quality of the cell (**Supplementary Figure S1A**). The degrees of association between nCount_RNA, nFeature_RNA, percent.MT, percent.HB, and percent.Ribosome were shown by correlation analysis (**Supplementary Figure S1B**). **Supplementary Figure S1C** displayed the integration of the three datasets after batch effect adjustment.

**Figure 1:**
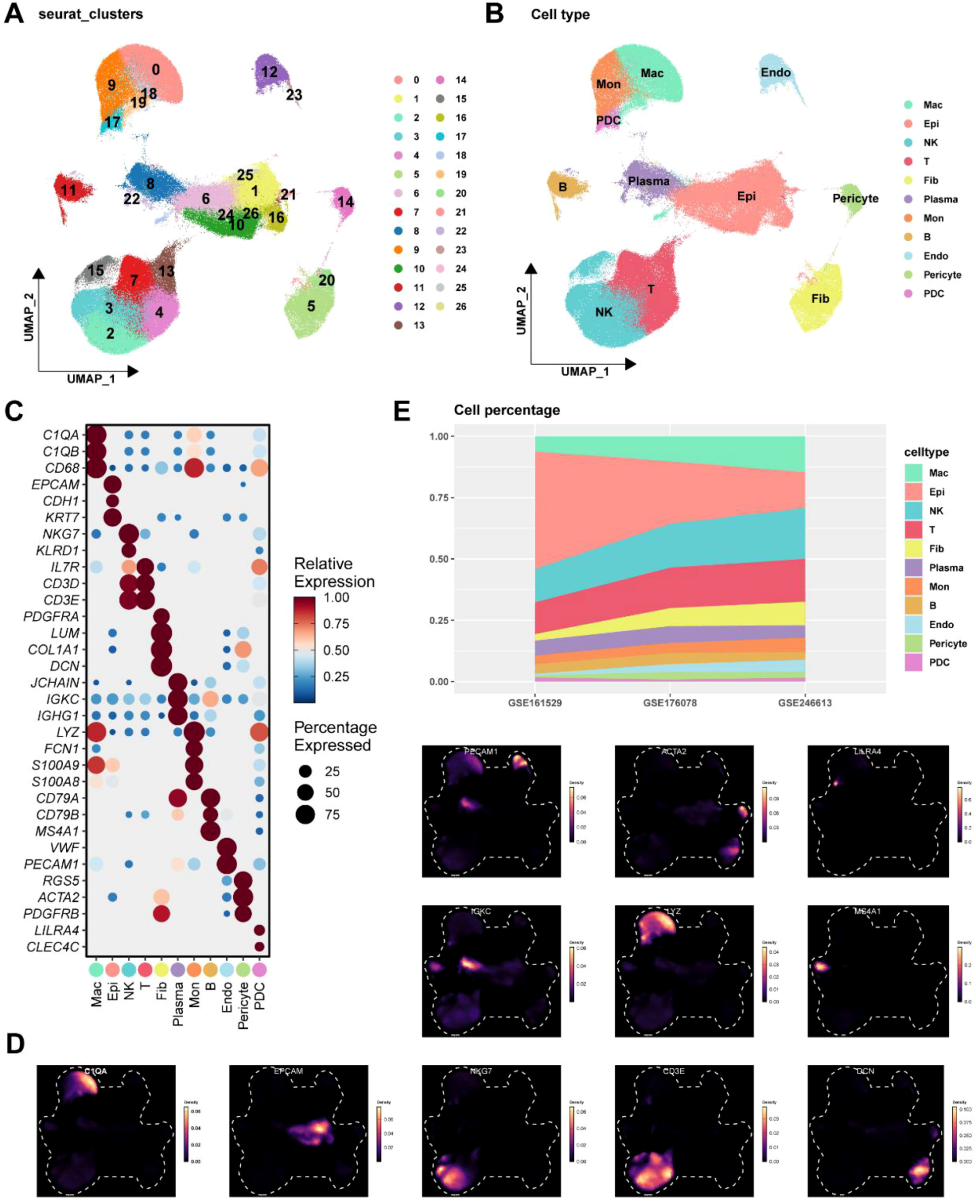
Classification and annotation of cell subpopulations in TNBC. **(A)** 27 cell clusters identified by Seurat in the UMAP plot. **(B)** 11 cell types annotated according to the expression of the marker genes. **(C)** The expression level of representative marker genes for cell subpopulations. **(D)** The expression density of representative marker genes for cell subpopulations. **(E)** Percentage of cells in the 11 cell subpopulations identified in GSE161529, GSE176078, and GSE246613.

UMAP analysis was employed to cluster scRNA-seq data from TNBC based on gene expression patterns, identifying a total of 27 distinct clusters (**Figure 1A**). Subsequently, classification annotation based on the expression profiles of commonly recognized marker genes identified 11 cell subpopulations: B cells, T cells, NK cells, plasma cells, monocytes, macrophages, plasmacytoid dendritic cells (pDCs), fibroblasts, endothelial cells, epithelial cells, and pericytes (**Figure 1B**). These included macrophages (*CD68*, *C1QA*, and *C1QB*), epithelial cells (*EPCAM*, *CDH1*, and *KRT7*), NK cells (*KLRD1* and *NKG7*), T cells (*CD3D*, *CD3E*, and *IL7R*), fibroblasts (*PDGFRA*, *COL1A1*, *DCN*, and *LUM*), plasma cells (*JCHAIN*, *IGKC*, and *IGHG1*), monocytes (*S100A8*, *S100A9*, *FCN1*, and *LYZ*), B cells (*MS4A1*, *CD79A*, and *CD79B*), endothelial cells (*VWF*, and *PECAM1*), pericytes (*RGS5*, *ACTA2*, and *PDGFRB*), and pDCs (*LILRA4* and *CLEC4C*) were all identified using the aforementioned markers (**Figure 1C**). Moreover, the density distribution profiles of representative marker genes for each cell subpopulation are presented in the UMAP plot (**Figure 1D**). More specifically, the proportions of the 11 identified cell subpopulations across each dataset were also shown in the **Figure 1E**.

### Expression patterns of SL activity in TNBC

**Figure 2:**
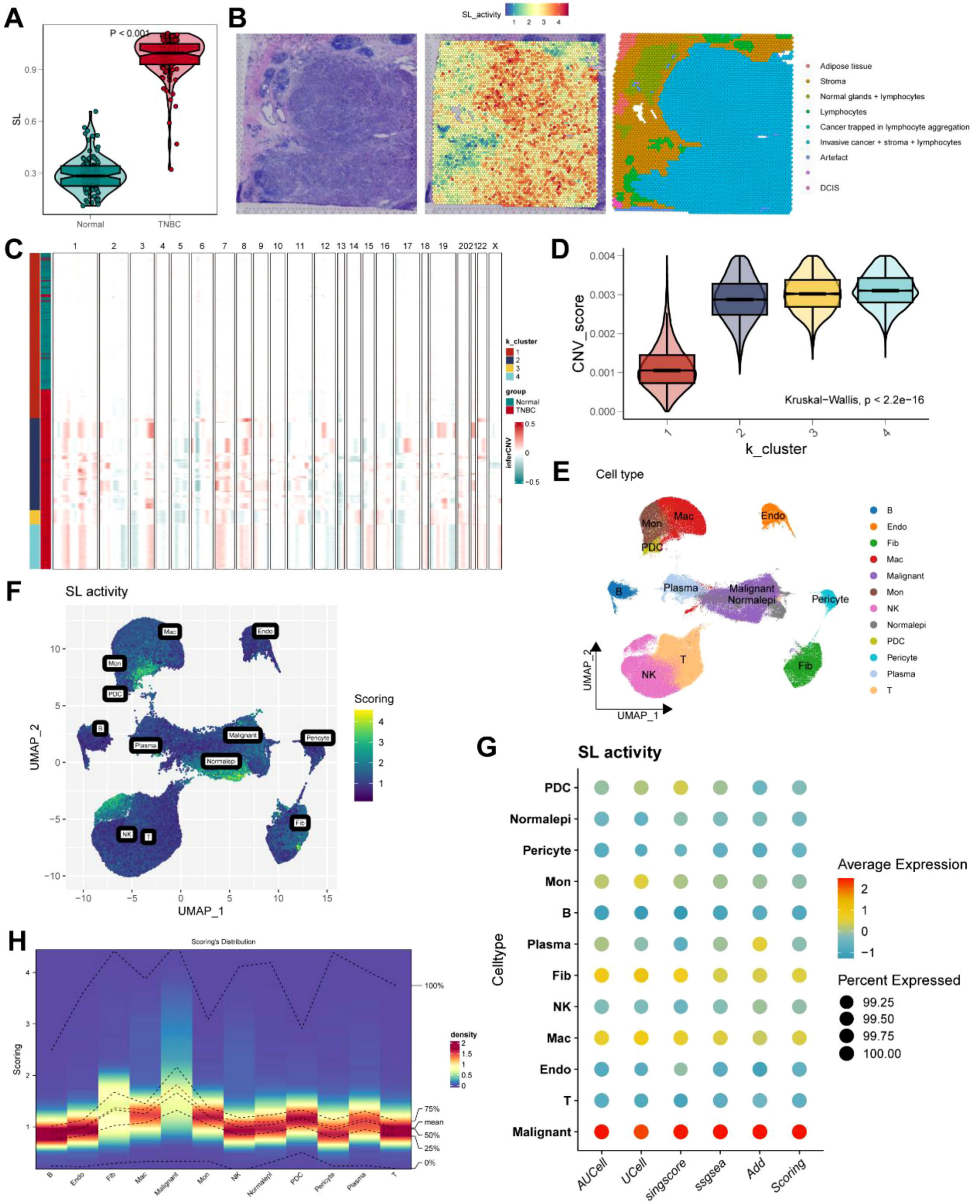
Evaluation of synthetic lethality (SL) activity in TNBC. **(A)** The expression difference of SL activity in the TCGA. **(B)** Spatial distribution characteristics of SL activity and TNBC. **(C)** Identification of TNBC malignant epithelial cells. **(D)** The level of CNV score between clusters by inferCNV. **(E)** The distribution of TNBC malignant cells and normal epithelial cells in UMAP plot. **(F)** The density of the SL activity within the TNBC microenvironment. **(G)** Dot plot showed the gene set scoring of SL activity across five algorithms. **(H)** SL scoring distribution across cell types in heatmap.

To elucidate the significant contribution of SL activity to the progression of TNBC, we conducted a comprehensive investigation into its expression patterns and distribution. First, within the TCGA cohort, patients were stratified according to the clinical phenotype, and our analysis revealed that individuals with TNBC experienced markedly elevated expression levels of SL activity compared to their counterparts (**Figure 2A****, P** < 0.001). However, given the heterogeneity of spatial organization and the complexity of the microenvironment within tissue structures, SL activity at the bulk RNA-seq expression level cannot yet provide insights into the state of individual tumor cells. ST was employed to detect the transcriptional status and spatial positioning of TNBC cells and their surrounding neighboring cells, thereby revealing the intracellular expression characteristics of SL activity within the core tumor region and exploring its relationship with the tumor. As shown in **Figure 2B**, TNBC cells in the HE-stained sections demonstrated infiltrative development with unclear margins, characterized by solid nests and sheet-like formations. The percentage of ductal development was modest, with invasive cancer+stroma+lymphocytes being the major components and occupying the biggest proportion. Furthermore, the SL activity expression level in this location was proportionally the greatest.

Gene expression information from individual cells was then obtained using scRNA-seq data in order to better describe the relationship between tumor cells and SL activity. In order to accurately evaluate cellular heterogeneity in gene expression, genetic variation, and other variables, we used inferCNV analysis to search for distinctive TNBC cells. Results revealed that epithelial cells were classified into four clusters, with clusters K2, K3, and K4 having considerably greater CNV scores than K1 (P < 0.001) (**Figures 2C, D**). It suggested that these clusters reflected malignant TNBC cells, whereas K1 likely had a fraction of normal epithelial cells intermingled inside (**Figure 2E**). Consistent with ST analysis, SL activity expression levels in the tumor region were significantly higher than those in normal epithelial cells (**Figure 2F**). Gene set scoring was employed to measure the activity level of SL gene sets in single-cell data, and five algorithms, including AUCell, UCell, singscore, ssGSEA, and AddModuleScore, exhibited considerable SL activity in TNBC malignant epithelial cells within tumor microenvironment cell subpopulations (**Figure 2G**). The density distribution of SL activity scoring across the TNBC microenvironment was clearly shown by the UMAP visualization, with the same observations as our appeal. (**Figure 2H**).

### Assessment of heterogeneity of SL activity in TNBC malignant cells

**Figure 3:**
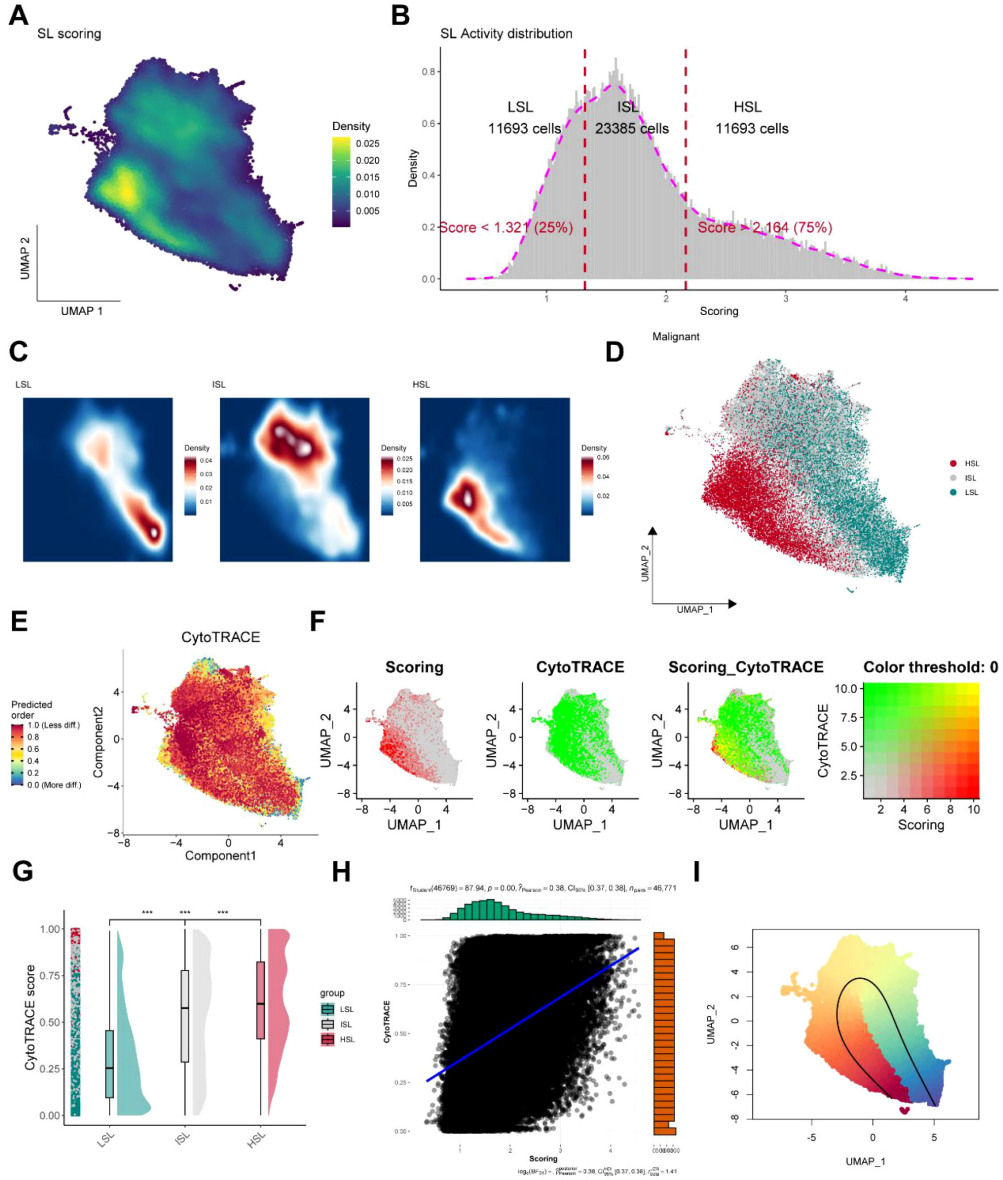
Synthetic lethality (SL) activity heterogeneity in TNBC malignant cells. **(A)** Density distribution of SL activity scores. **(B)** SL activity distribution in TNBC malignant cells. **(C)** Density distribution characteristics of the HSL, ISL, and LSL cells. **(D)** Spatial expression characteristics of the HSL, ISL, and LSL cells. **(E)** CytoTRACE analysis for the prediction of differentiation potential. **(F)** The co-expression relationship of SL activity and CytoTRACE score. **(G)** CytoTRACE scoring differences among the HSL, ISL, and LSL groups. **(H)** Correlation analysis for SL activity and CytoTRACE score. **(I)** Slingshot inference trajectory for TNBC.

Although we observed uneven distribution of SL activity within the TNBC microenvironment, its heterogeneity within malignant cells remains unclear. Gene set scoring for SL activity revealed a distinct tendency in density distribution, with significant differences in expression levels among malignant cells (**Figure 3A**). Subsequently, we selected a total of 46,771 malignant cells identified by inferCNV analysis for CytoTRACE analysis to delineate heterogeneous features within malignant cell subpopulations. Cells were stratified by applying 75th and 25th percentile thresholds to distinguish SL score levels, ultimately yielding 11,693 high SL activity cells (HSL) with an SL score of more than 2.164 (75%), 11,693 low SL activity (LSL) cells with an SL score of less than 1.321 (25%), and 23,385 intermediate SL activity (ISL) cells with SL scores distributed between the two groups (**Figure 3B**). Moreover, the three groups HSL, ISL, and LSL exhibited distinct density distribution patterns, with HSL showing a significantly clustered expression pattern in malignant TNBC cells (**Figures 3C, D**). CytoTRACE analysis revealed that malignant cell subpopulations throughout TNBC exhibited consistently elevated CytoTRACE scores, with HSL-distributed regions demonstrating the highest levels. A significant negative correlation was observed between CytoTRACE scores and cellular developmental potential, suggesting that HSL-associated cell subpopulations possess higher stemness levels, reduced differentiation capacity, and increased malignant progression (**Figures 3E-G**). Correlation analysis further confirmed a strong association between SL and CytoTRACE scores (**Figure 3H**, r = 0.38). Additionally, we determined that the HSL cell subpopulation in TNBC may serve as a starting point for differentiation trajectories based on the developmental potential score determined by CytoTRACE. A potential malignant differentiation route, where tumor development shifts from high to low SL activity, was deduced by slingshot analysis. For TNBC patients, this heterogeneity in SL activity offers novel therapeutic strategies (**Figure 3I**).

### Intercellular communication in the TNBC microenvironment

**Figure 4:**
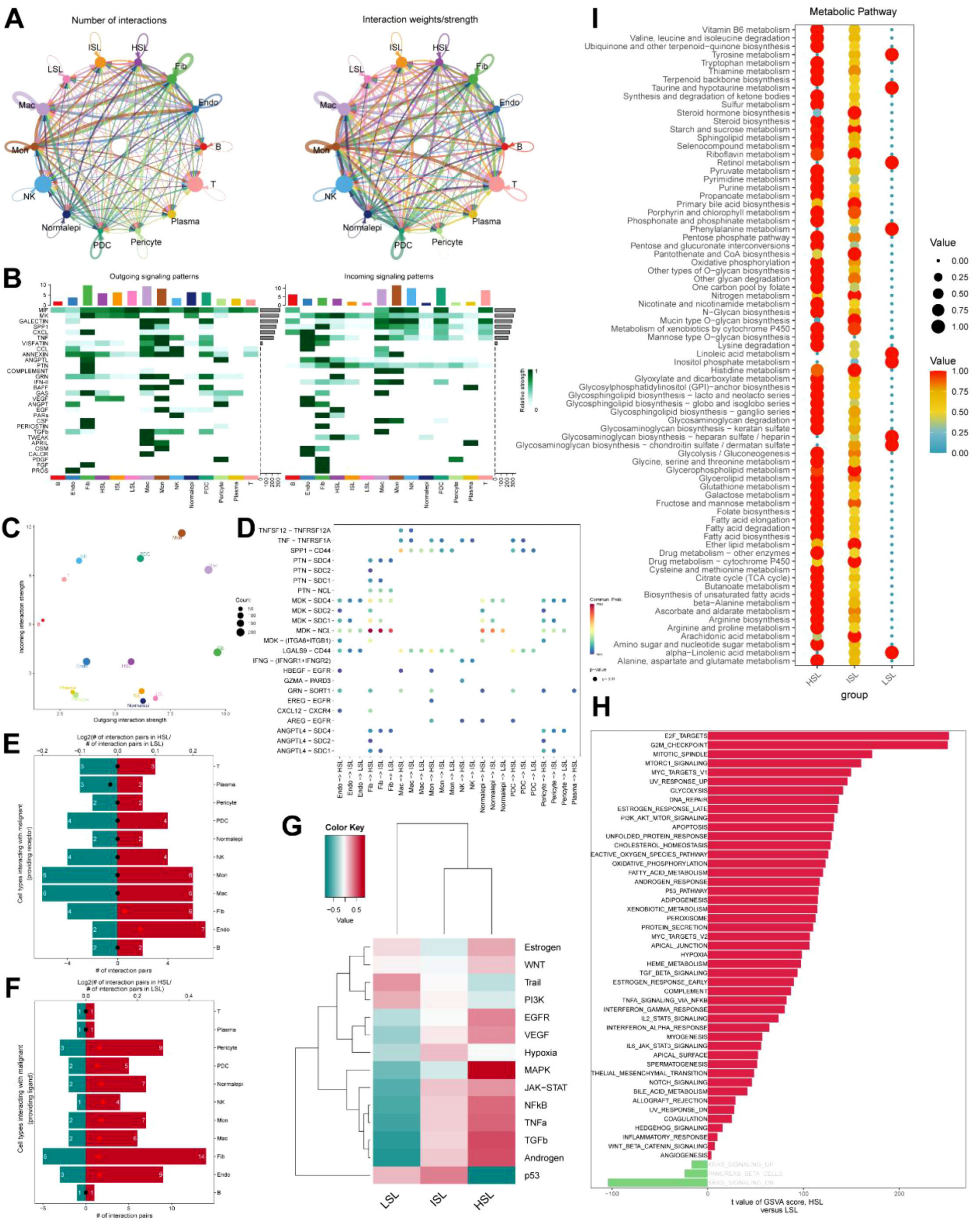
Cell communication and signaling pathway analysis. **(A)** TNBC tumor microenvironment intercellular communication networks. **(B)** Patterns of outgoing and incoming signaling pathways in TNBC. **(C)** Communication patterns between outgoing and incoming pathways. **(D)** Ligand-receptor interaction analysis of HSL, ISL, and LSL cells. **(E, F)** Interaction pairs of cell types interacting with HSL and LSL cells in providing receptor and ligand. **(G)** Pathway responsive for activity inference in SL activity groups. **(H)** GSVA analysis for HSL and LSL cells. **(I)** Metabolic pathway enrichment analysis in SL activity groups.

Malignant HSL activity epithelial cells in the TNBC microenvironment were shown to participate in a high number of contacts with higher weight and strength than LSL malignant cells, responding to research on their intercellular communication (**Figure 4A**). According to further study on the signaling network concerning malignant HSL epithelial cells, the major genes in outgoing signaling patterns were *MK*, *MIF*, *VEGF*, and *ANNEXIN*, whereas the main genes in incoming signaling patterns were *TWEAK*, *EGF*, *GRN*, *PTN*, and *MK* (**Figure 4B**). Moreover, malignant LSL cells were shown to have a larger outgoing interaction pathway than malignant HSL cells in relation to contact intensity, despite the incoming interaction pathway being the opposite (**Figure 4C**). An examination of ligand-receptor interactions revealed that *MDK* played a critical role in regulating the signaling connections between the SL activity and the TNBC microenvironment, which drove to the advancement of the disease (**Figure 4D**). In terms of receptor-ligand provision, HSL malignant cells exhibit significantly more contact pairing with other cell subpopulations among the TNBC microenvironment compared to LSL malignant cells (**Figures 4E, F**). In particular, the *MAPK*, *JAK-STAT*, *TGFβ*, and androgen pathways were shown to be significantly active tumor-associated signaling pathways in HSL malignant cells using PROGENY research. On the other hand, only some pathways, such *p53*, showed activity in LSL malignant cells. This suggested that in TNBC, SL activity levels were strongly correlated with tumor malignant progression, consistent with its heterogeneous characteristics (**Figure 4G**). Furthermore, GSVA analysis revealed that compared to the LSL malignant cell subpopulation, HSL malignant cells exhibited enrichment in numerous pathways, with more pronounced enrichment observed in signaling pathways such as E2F_TARGETS, G2M_CHECKPOINT, MYC_TARGETS_V1, and DNA_REPAIR, which are relevant to targeted and immunotherapy (**Figure 4H**). One of the hallmarks of cancer and progression is metabolic change. We examined variations in metabolic pathway activity across the three cell subpopulations using the scMetabolism algorithm. The HSL group showed broad expression of many pathways, including drug metabolism, fatty acid metabolism, glycolysis, and other activities, in contrast to the LSL group (**Figure 4I**). The aforementioned research findings indicated a close association between SL activity and the development of TNBC. Further investigation will advance its clinical application.

### SL activity-driven signature gene identification

**Figure 5:**
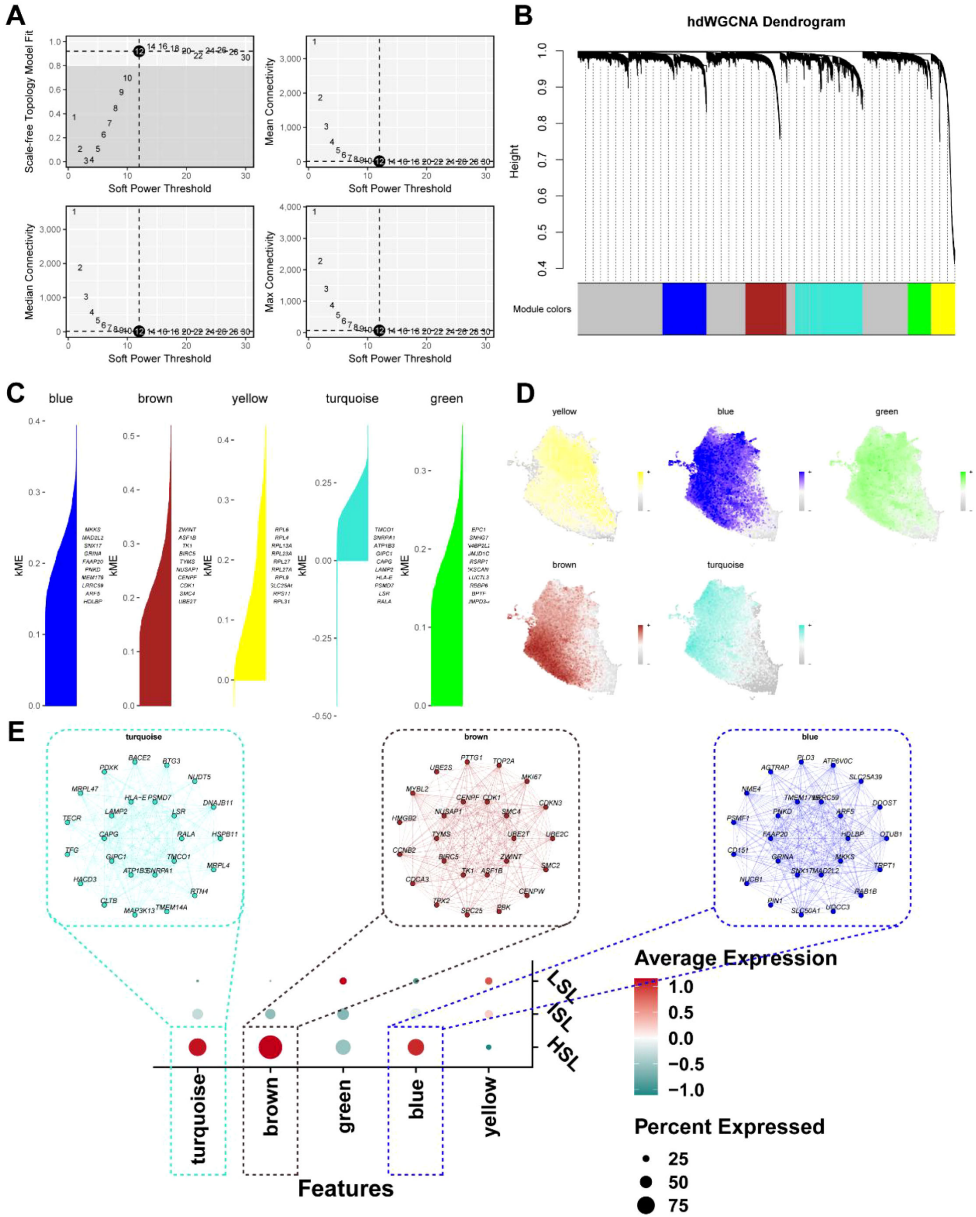
HdWGCNA identificated hub genes associated with high synthetic lethality (HSL) cells. **(A)** Selection of soft-thresholding power and topology fit index for network construction. **(B)** Dendrogram for module clustering in hdWGCNA. **(C)** Top 10 module-specific genes. **(D)** The distribution of module-specific genes associated with HSL cells. **(E)** An examination of module-trait associations revealed modules enriched in the HHM group.

High-dimensional weighted gene co-expression network analysis (hdWGCNA) was employed to decipher the gene co-expression network framework within scRNA-seq data, identifying gene modules highly correlated with HSL subpopulations. By setting the optimal soft threshold to 12 and the topology fit index to 0.9, a scale-free co-expression network was constructed, identifying five distinct gene co-expression modules: blue, brown, yellow, turquoise, and green (**Figures 5A, B**). Module saliency analysis revealed the top 10 genes within each module, along with unique expression patterns and connectivity structures between modules (**Figures 5C, D**). Among these, the blue, brown, and turquoise modules showed high correlation within the HSL group, with expression levels significantly higher than other modules (**Figure 5E**). The 300 hub genes contained within these modules are detailed in **Supplementary Table S5**.

**Figure 6:**
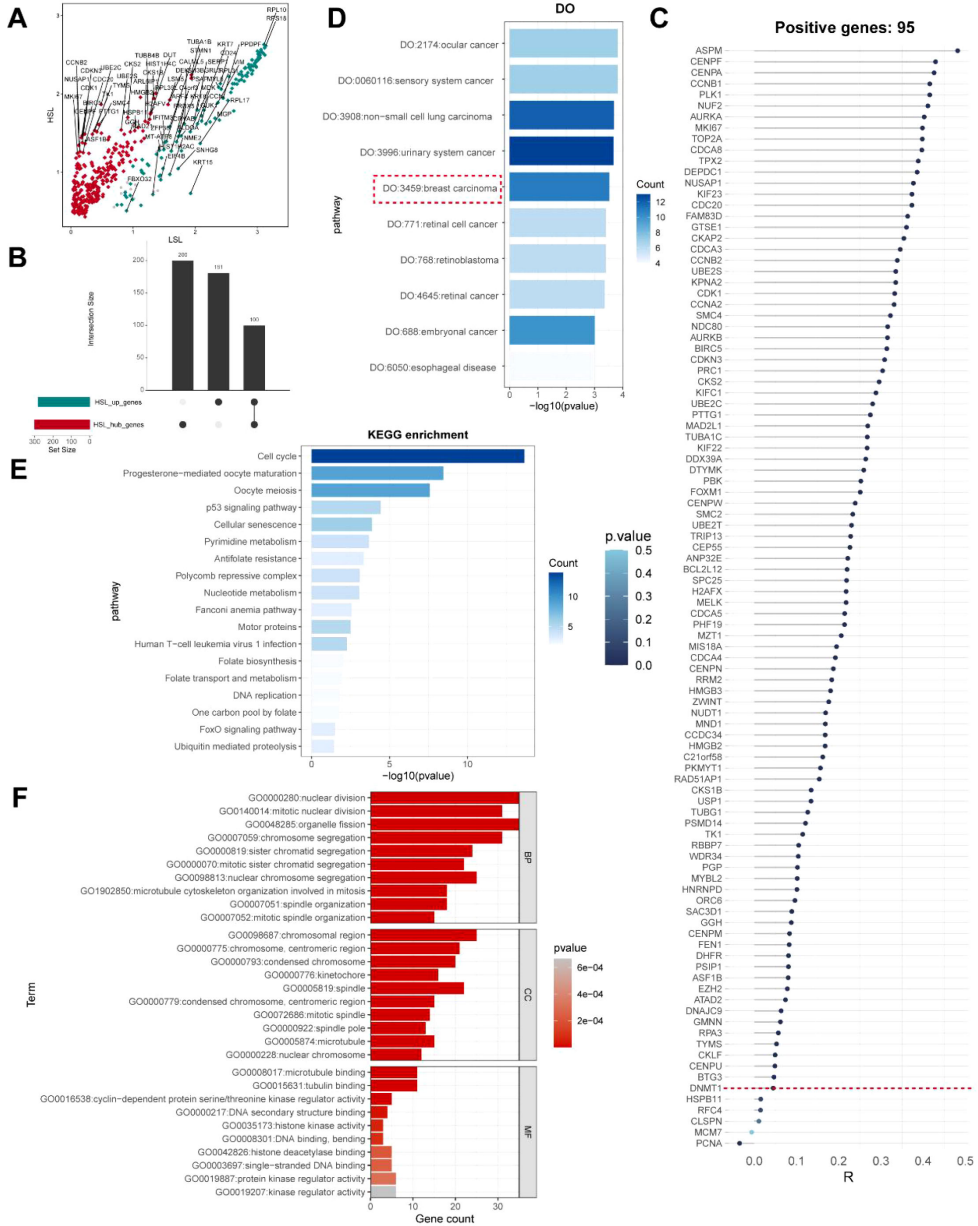
Identification and enrichment analysis of hub genes for driving high synthetic lethality (HSL) activity. **(A)** DEG analysis between SL activity groups. **(B)** Venn diagram for intersecting genes from hdWGCNA and differential expression analysis. **(C)** Pearson correlation analysis found positively genes linked to HSL activity. **(D)** DO enrichment analysis. **(E)** KEGG enrichment analysis. **(F)** GO enrichment analysis.

Subsequently, we performed differential expression analysis to identify genes associated with HSL cells that drive transcriptional alterations in TNBC. A total of 281 upregulated differentially expressed genes were identified (**Figure 6A**; **Supplementary Table S6**). Following hdWGCNA crossing to get the module’s hub genes and the DEGs that were up-regulated in the HSL malignant cells, we ultimately discovered 100 crosstalk genes that were responsible for the HSL activity (**Figure 6B**; **Supplementary Table S7**). Pearson correlation analysis further identified the features most strongly associated with SL activity, pinpointing 95 signature genes (**Figure 6C**). Subsequently, enrichment analysis was performed to investigate the biological roles that these 95 distinctive genes were implicated in. Initially, it was discovered by DO analysis that these genes were substantially linked to a number of cancers, including breast cancer (**Figure 6D**; **Supplementary Table S8**). These genes had strong relationships with the p53 signaling pathway, DNA replication, and metabolism-related pathways, according to the findings of KEGG enrichment analysis (**Figure 6E**; **Supplementary Table S9**). Terms including nuclear division (GO: 0000280), mitotic nuclear division (GO: 0140014), and organelle fission (GO: 0048285) were shown to be highly enriched in biological processes by GO enrichment analysis (**Figure 6E**, **Supplementary Table S10**). Genes associated with the chromosomal region (GO: 0098687) and condensed chromosome (GO: 0000793) were significantly enriched in cellular components, whereas the molecular functions of microtubule binding (GO: 0008017), tubulin binding (GO: 0015631), and histone kinase activity (GO: 0035173) were remarkably enriched.

### Hallmark gene selection describing HSL activity

**Figure 7:**
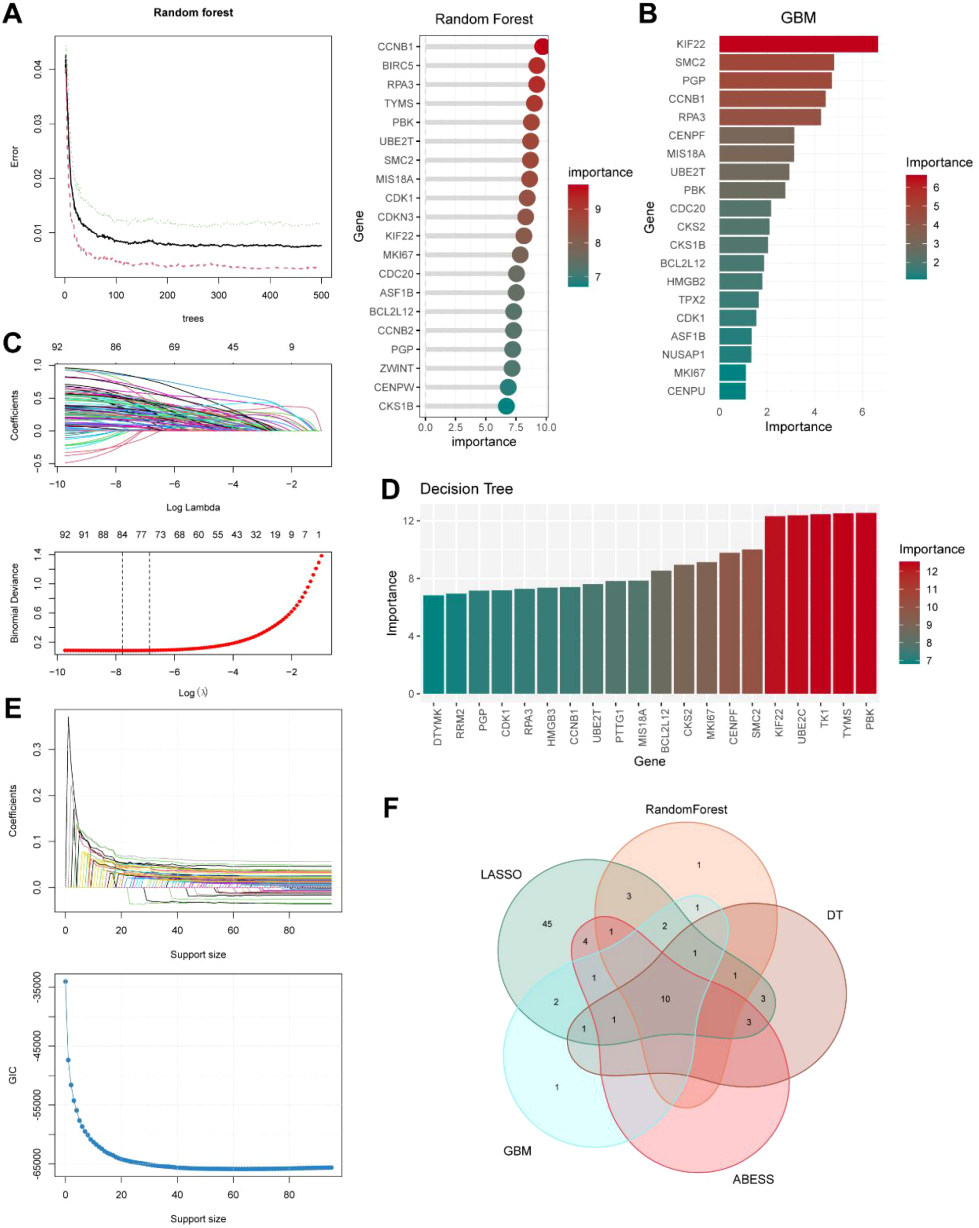
Identification of the hallmark genes associated with high synthetic lethality (HSL) activity. **(A)** Random Forest algorithm screened for characteristic genes. **(B)** GBM algorithm screened for characteristic genes. **(C)** LASSO algorithm screened for characteristic genes. **(D)** Decision Tree algorithm screened for characteristic genes. **(E)** ABESS algorithm screened for characteristic genes. **(F)** The ten hallmark genes that the five algorithms shared were shown in a Venn diagram.

We used a combination of machine learning methods, including Random Forest, GBM, LASSO, Decision Tree, and ABESS for feature selection, to accurately identify marker genes linked to HSL activity in TNBC malignant cells. The level of importance was used to determine the top 20 signature genes for Random Forest (**Figure 7A**; **Supplementary Table S11**). The GBM algorithm was then used to identify 20 signature genes, which were also rated by importance, similar to Random Forest (**Figure 7B**; **Supplementary Table S12**). 78 prognostic signature genes were found using Lasso after 10-fold cross-validation (**Figure 7C**; **Supplementary Table S13**). Following that, 20 signature genes were found using Decision Tree and ABESS, respectively (**Figures 7D, E**; **Supplementary Tables S14**, **S15**). Ten signature genes were ultimately identified by crossover amongst the five machine learning algorithms: *PGP*, *KIF22*, *CCNB1*, *RPA3*, *BCL2L12*, *SMC2*, *MKI67*, *PBK*, *CDK1*, and *MIS18A* (**Figure 7F**).

### Expression and validation of hallmark genes

**Figure 8:**
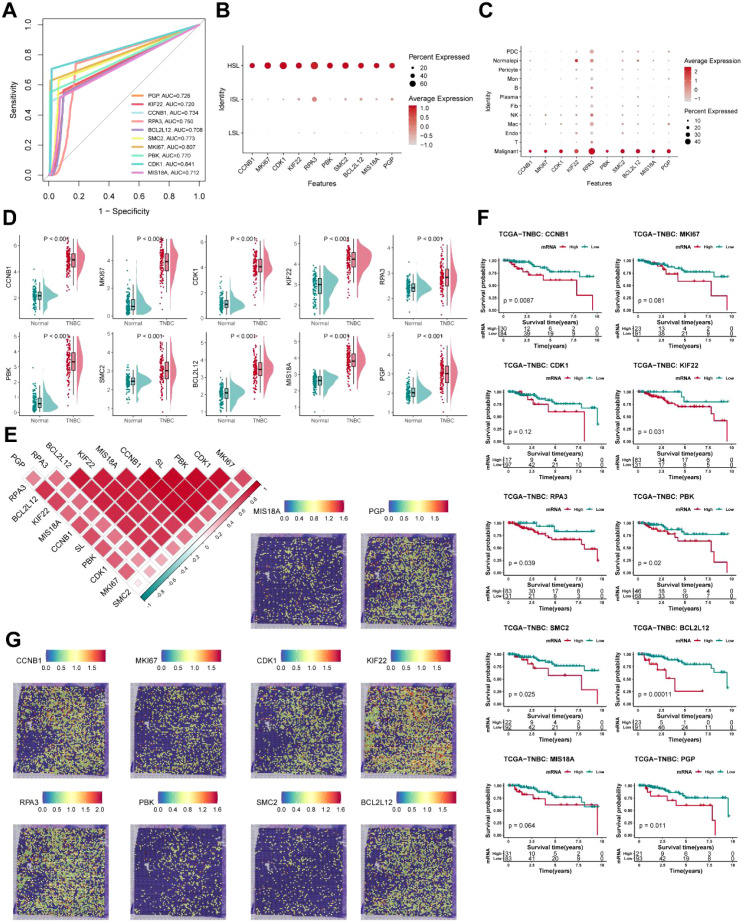
Assessment of expression and prognosis of hallmark genes. **(A)** Kaplan-Meier survival analysis verified the accuracy of hallmark genes. **(B)** Expression of hallmark genes in HSL, ISL, and LSL malignant cells. **(C)** Expression of hallmark genes across all cell subpopulations in the TNBC microenvironment. **(D)** Expression of hallmark genes in the TCGA cohort. **(E)** Correlation analysis for SL activity and hallmark genes. **(F)** Kaplan-Meier survival analysis of hallmark genes in the TCGA cohort. **(G)** Spatial transcriptomics analysis of 10 hallmark genes in the TNBC specimen tissue.

After integrating five machine learning algorithms, we ultimately identified ten hallmark genes. To further evaluate the accuracy of these genes in guiding prognosis, we first assessed their predictive capabilities using ROC curve analysis in scRNA-seq data. Results indicated that all 10 hallmark genes exhibited relatively high areas under the curve (AUC), specifically as follows: *PGP* (AUC = 0.726), *KIF22* (AUC = 0.720), *CCNB1* (AUC = 0.734), *RPA3* (AUC = 0.750), *BCL2L12* (AUC = 0.708), *SMC2* (AUC = 0.773), *MKI67* (AUC = 0.807), *PBK* (AUC = 0.770), *CDK1* (AUC = 0.841), and *MIS18A* (AUC = 0.712) (**Figure 8A**). Furthermore, the expression levels of these genes were closely associated with SL activity, exhibiting significant expression in HSL malignant cells while showing markedly reduced expression in ISL and LSL malignant cells (**Figure 8B**). Within the broader TNBC microenvironment, we observed that these marker genes were primarily expressed in malignant tumor cells, with some genes showing partial or low expression in normal epithelial cells and macrophages (**Figure 8C**). Subsequent validation of the bulk RNA-seq level confirmed similar results, with expression levels of 10 hallmark genes significantly higher in TNBC than in normal tissue (**Figure 8D**). Correlation analysis also indicated that the expression levels of these genes were closely associated with SL activity (**Figure 8E**). Next, we performed Kaplan-Meier survival analysis to evaluate survival difference in the TCGA cohort, dividing patients into high- and low-expression groups based on the optimal cutoff value for each hallmark gene. Analysis revealed that higher expression levels of these genes were associated with poorer overall survival (OS), with *PGP*, *KIF22*, *CCNB1*, *RPA3*, *BCL2L12*, *SMC2*, and *PBK* significantly correlated with poor prognosis in TNBC patients (*p* < 0.05, **Figure 8F**). Survival analysis results for these genes in other datasets, including METABRIC, GSE58812, and GSE31519, were presented in **Supplementary Figure S2**. It is notable that *SMC2* shows opposite survival associations between METABRIC and GSE58812. We speculate that this difference may stem from cohort heterogeneity, endpoint/follow-up and treatment differences, as well as KM instability caused by small sample sizes and group imbalances. At the ST level, *PGP*, *KIF22*, *CCNB1*, *RPA3*, *BCL2L12*, *MKI67*, and *CDK1* exhibited diffuse expression throughout TNBC tumor tissues, with high expression in tumor core regions. This pattern strongly overlapped with SL activity distribution areas, underscoring the accuracy of the selected genes (**Figure 8G**).

### Benchmarking of the machine learning model and SHAP analysis of hallmark genes

**Figure 9:**
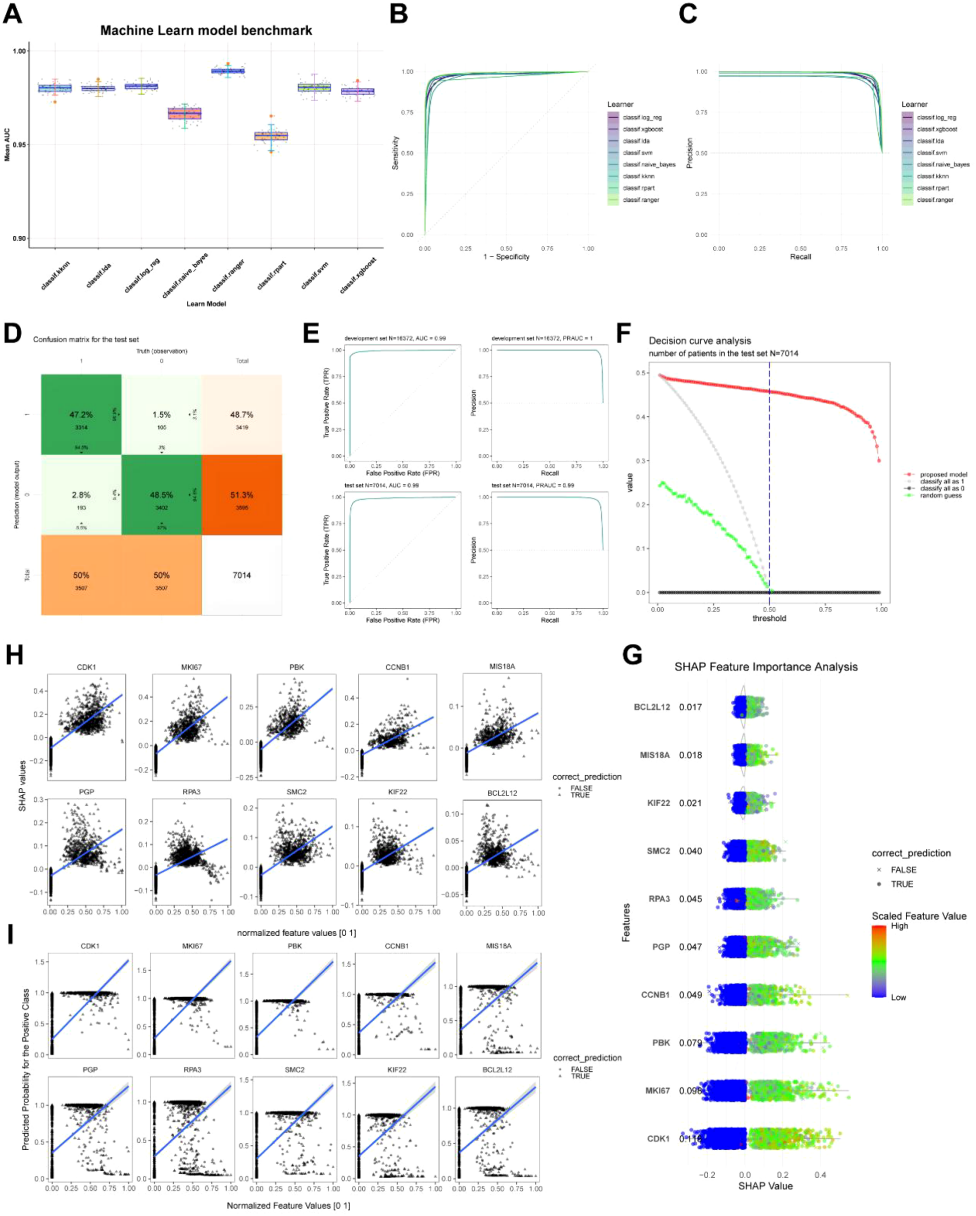
Construction of the optimal model and SHAP explanation for feature. **(A)** Benchmarking of learners for performance assessment. **(B)** Receiver-operating Curve curves examined the accuracy of classifiers. **(C)** Precision-recall curves examined the accuracy of classifiers. **(D)** The confusion matrix of the test set with the Benchmarking. **(E)** ROC AUC and PRAUC of development and test sets with the Benchmarking. **(F)** Decision curve analysis for clinical applicability assessment. **(G)** SHAP analysis revealed feature genes contribution. **(H)** Correlation between SHAP values and the expression of hallmark genes. **(I)** Correlation between predicted probability for the positive class and the expression of hallmark genes.

Even though we used five machine learning algorithms to identify ten signature genes, it was still unknown how well these models’ function, and the best model had not yet been discovered. Through benchmarking which was employed to fairly compare the performance of multiple algorithms on the same task, we were able to establish a standardized evaluation framework with eight learners, including KNN, NB, Ranger, RPART, SVM, Log_reg, LDA, and XGBoost, for contrasting each machine learning model’s accuracy and effectiveness. With the greatest ROC AUC (Receiver Operating Characteristic Area Under the Curve) and PRAUC (Precision-Recall Area Under the Curve), the Random Forest model was shown to have the best performance and stability (**Figures 9A-C**; **Supplementary Table S16**). The confusion matrix of the test set indicated that the Random Forest algorithm achieved a prediction accuracy of 94.5% and a precision of 96.9% in identifying HSL-related genes (**Figure 9D**). Its ROC AUC and PRAUC values in the test set were both 0.99, demonstrating high robustness (**Figure 9E**). Furthermore, decision curve analysis revealed that this model provided more significant net clinical benefit (**Figure 9F**). In summary, the Random Forest model might serve as the optimal algorithm in this study for identifying feature genes associated with HSL activity.

SHAP analysis could integrate into supervised machine learning models to enhance prediction credibility by intuitively visualizing each feature’s overall contribution to model output through SHAP value. Features were then ranked based on average absolute SHAP values and visualized to show their distribution across different prediction outcomes (**Figure 9G**). Each hallmark gene exhibited a clear correlation between its SHAP value and the predicted probability for the positive class (**Figures 9H, I**).

### The synergistic effect of *KIF22* and *KRAS in vivo* and *in vitro*

**Figure 10:**
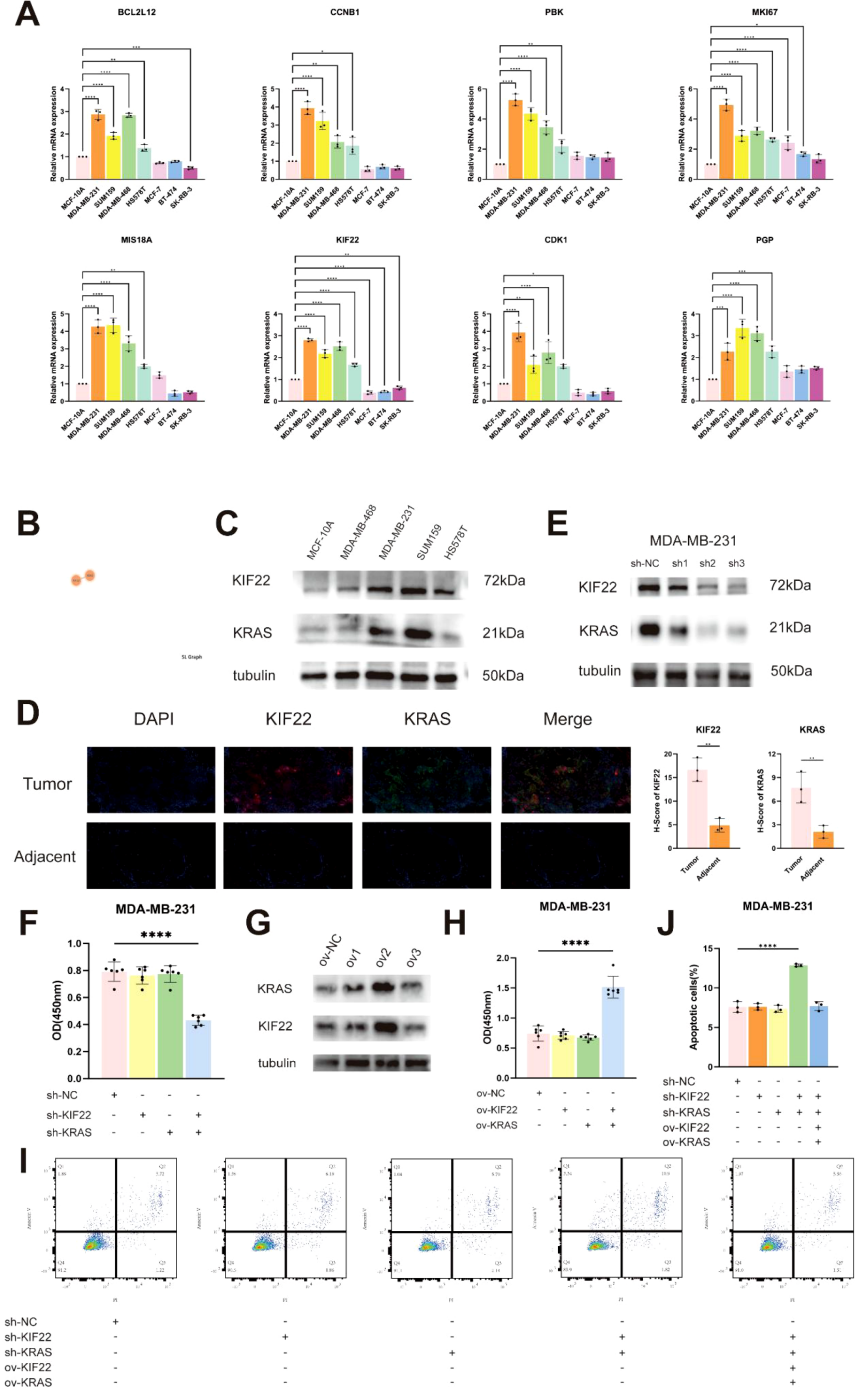
Simultaneous loss of *KIF22* and *KRAS* led to a slowdown in cell proliferation and an increase in apoptosis. **(A)** The mRNA expression levels of *KIF22* and *KRAS* in breast cancer cells (n = 3). **(B)** The SynLethDB database identifies *KRAS* as a synthetic lethal partner for *KIF22*. **(C)** The protein expression levels of *KIF22* and *KRAS* in TNBC cells. **(D)** mIHC illustrates the expression and localization of *KIF22* and *KRAS* in TNBC tissues (n = 3). **(E)** The knockdown efficiency of lentivirus targeting *KIF22* and *KRAS*. **(F)** CCK-8 assays assess the impact of *KIF22/KRAS* knockdown on cell proliferation. (n = 6) **(G)** The overexpressing efficiency of lentivirus targeting *KIF22* and *KRAS*. **(H)** CCK-8 assays to examine the effects of overexpressing *KRAS/KIF22* (n = 6). **(I, J)** Research on transfection inducing apoptosis (n = 3). *p < 0.05, **p < 0.01, ***p < 0.001, ****p < 0.0001.

First, we conducted RT-qPCR detection on these ten genes in various breast cancer cell lines (BCCLs) including TNBC and non-TNBC and normal breast epithelial cell lines. It was found that the mRNA levels of these ten genes in TNBC cell lines (MDA-MB-231, SUM159, Hs 578t and MDA-MB-468) were higher than other cell lines (**Figure 10A**). According to **Supplementary Figure S2**, *KIF22* and *SMC2* are the only two genes significantly associated with prognosis. After consulting the materials, we believe that *KIF22* is of greater research value, so we chose it as the research object. According to the SynLethDB database, we learned that *KRAS* is the sole synthetic lethal pair of *KIF22* (**Figure 10B**). Therefore, we detected the protein expression levels of *KIF22* and *KRAS* in multiple TNBC cell lines. The results indicated that the protein expression of these two genes in TNBC cell lines was higher than normal cell lines (**Figure 10C**). Subsequently, the protein expression and localization of *KIF22* and *KRAS* in adjacent tissue samples and TNBC tissue samples were observed by multiplex immunofluorescence histochemical method. Immunofluorescence staining further observed that *KIF22* and *KRAS* are respectively located in the nucleus and cytoplasm. We distinguished tumor cells from interstitial cells by panCK staining and calculated the IHC score. The results showed that the expressions of *KIF22* and *KRAS* in cancer tissues were much higher than those in adjacent tissues (**Figure 10D**).

To investigate the functions of *KIF22* and *KRAS* in TNBC, we knocked them down in MDA-MB-231 cells respectively (**Figure 10E**). When *KIF22* or *KRAS* was knocked down alone, there were no signs of increased apoptosis or slowed proliferation in MDA-MB-231 cell. However, when we simultaneously knocked down *KIF22* and *KRAS*, there was a relatively significant slowdown in proliferation and an increase in apoptosis (**Figures 10F, I, J**). In MDA-MB-231 cells with simultaneous knockdown of *KIF22* and *KRAS*, we simultaneously overexpressed *KIF22* and *KRAS*. We found that the cell proliferation rate recovered. However, no changes were found in MDA-MB-231 cells that overexpressed *KIF22* and *KRAS* separately (**Figure 10H**).

**Figure 11:**
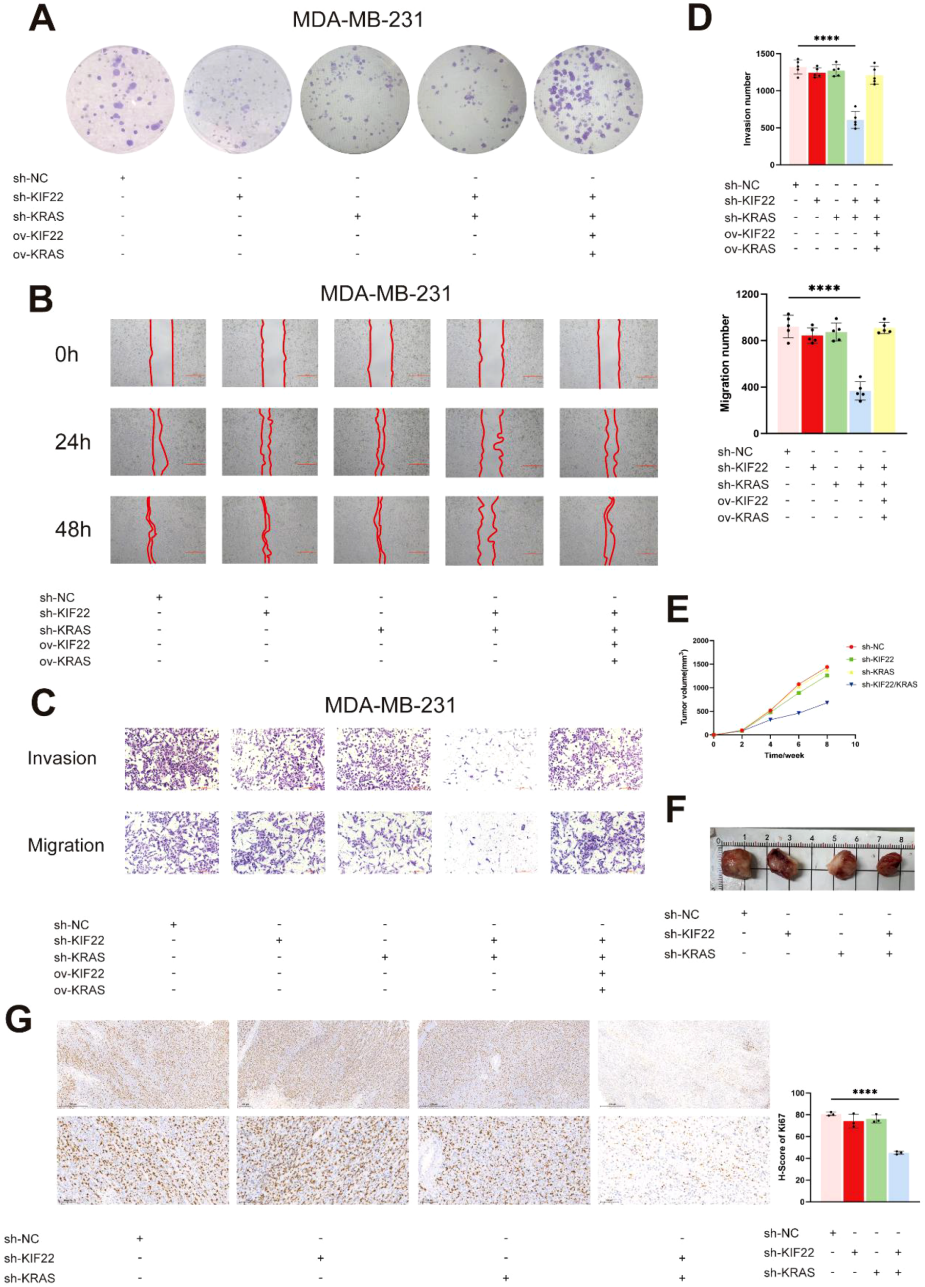
The effects of KIF22 and KRAS knockdown on the biological functions of triple-negative breast cancer (TNBC) cells and tumor growth *in vivo*. **(A)** Colony formation assay was performed on MDA-MB-231 cells with different transfection treatments. Crystal violet staining was used to visualize colonies. **(B)** Wound healing assay was conducted to evaluate cell migration capacity. Scratches were made on confluent cell monolayers, and images were captured at 0 h, 24 h, and 48 (h) **(C)** Transwell invasion assay and migration assay were used to assess cell invasive and migratory potentials. **(D)** Quantitative analysis of migrated/invaded cells in Transwell assays using ImageJ software (n = 5). Data are presented as mean ± standard deviation, and statistical significance was determined by appropriate statistical tests (****P < 0.0001). **(E)** Transfected cell lines were injected into mice. Regularly monitor the tumor volume. **(F, G)** Immunohistochemical (IHC) staining of Ki-67 in tumor tissues (n = 3). *p < 0.05, **p < 0.01, ***p < 0.001, ****p < 0.0001.

Then, we conducted clone formation experiments (**Figure 11A**), wound healing experiments (**Figure 11B**) and TRANSWELL experiments (**Figure 11C**) on MDA-MB-231 cells. And the cells in the TRANSWELL assay results were counted using image j software (**Figure 11D**). When *KIF22* and *KRAS* were knocked down alone, as well as when both *KIF22* and *KRAS* were knocked down simultaneously. It was found that knocking down *KRAS* and *KIF22* alone had a relatively small or even no effect on the proliferation and migration abilities of cells, while knocking down both had a greater impact. Finally, we separately knocked down *KIF22* and *KRAS* in MDA-MB-231 cells, simultaneously knocked down *KIF22* and *KRAS*. We then inoculated them into nude mice to observe the tumor formation. The tumor volume of the mice was measured weekly (**Figure 11E**). At the 8th week after vaccination, the mice were sacrificed and the size of their tumors was measured (**Figure 11F**). It was found that mice with simultaneous knockdown of *KIF22* and *KRAS* had the slowest increase in tumor volume and the smallest tumors. There was no significant difference between the *KIF22* or *KRAS* group alone and the control group, and the growth rate was relatively fast. Finally, immunohistochemistry was performed on the tumors of four mice, and it was found that *KI67* was the lowest in the tumors with double knockdown, while there was little difference in the other three mice (**Figure 11G**).

## Discussion

TNBC is an aggressive subtype of breast cancer with a poor prognosis, characterized by negative expression of *ER*, *PR*, and *HER2* (35). Due to the lack of these targets, traditional targeted therapies have limited efficacy, making the development of new treatment strategies crucial (36). However, the emergence of SL strategies has made it possible to use the unique genetic defects or metabolic abnormalities of tumor cells for precision treatment of tumors. Research indicates that many patients with TNBC harbor *BRCA1/2* gene mutations or functional defects, leading to impaired DNA repair mechanisms (37). SL exploits these tumor-specific genetic vulnerabilities by inhibiting the activity of related biomolecules, thereby preventing the repair of DNA damage and ultimately inducing tumor cell death. In-depth research into the biological functions and molecular characteristics of SL facilitates the discovery of novel SL interactions and associated biomarkers, thereby expanding the pool of therapeutic targets for cancer treatment and enabling true precision medicine.

In this work, we first used single-cell RNA sequencing to provide a thorough examination of the tumor microenvironment landscape in TNBC. This further clarified the intricate cellular communication and interactions inside the TNBC microenvironment by revealing the spatial expression, biological roles, and diverse features of SL within malignant tumor cells. Based on SL activity, we were able to determine the developmental origins and deduce a developmental trajectory of TNBC cells using CytoTRACE and Slingshot analyses. Ten driver genes (*PGP*, *KIF22*, *CCNB1*, *RPA3*, *BCL2L12*, *SMC2*, *MKI67*, *PBK*, *CDK1*, and *MIS18A*) influencing SL activity were found by combining hdWGCNA with five machine learning algorithms. This provided a stratification technique for identifying individuals who would benefit from targeted treatment. The precise contribution of each characteristic to the prediction results was measured using SHAP analysis. The creation of biomarkers and innovative SL treatment approaches is facilitated by this interpretable gene significance ranking, which also improves practical applicability and gives TNBC patients fresh hope for treatment effectiveness and survival.

Cancer stem cells (CSCs) represent a subpopulation within tumors that possess self-renewal and multipotent differentiation capabilities (38). They resist cell death through multiple mechanisms, including overexpression of anti-apoptotic proteins, expression of multidrug resistance transporters, and activation of the *PI3K/Akt/mTOR* signaling pathway. These properties are considered the primary drivers of tumor recurrence and metastasis (39–41). Prior research by Blanco S et al. indicated that CSCs exhibited lower translational activity compared to differentiated mature cancer cells (42). However, subsequent studies proposed that high translational activity was a key factor driving CSC differentiation (43, 44). HSL cells, as a newly identified population, exhibited higher CytoTRACE scores, indicating a stronger stem-like state and lower differentiation potential. This suggested that HSL cells in TNBC might represent a type of CSC undergoing a transitional state from tumor stemness to fully differentiated malignant cells. In TNBC, CSCs research indicated their proportion was significantly higher than in other breast cancer subtypes. CSCs were considered a key factor contributing to the poor prognosis of TNBC, being closely associated with chemotherapy resistance, recurrence, and metastasis (45). Within TNBC, CSCs exhibited shared molecular mechanisms with TNBC cells, including metabolic plasticity, signaling pathways, and transcription factor regulation, thereby promoting TNBC progression (46, 47). Given the close biological and clinical association between CSCs and TNBC, targeted therapeutic strategies directed at CSCs hold promise for providing novel therapeutic approaches for TNBC treatment.

Additionally, a single-cell-level predictive model designed to identify HSL activity at the cellular level identified ten marker genes. Through in-depth literature search, we elaborate on *KIF22* and its’ synthetic lethal effects on *KRAS* based on the weights of SHAP values in TNBC.

It is well known that the Kinesin Family member drives the progression of mitosis (48). The protein encoded by *KIF22* is a member of the kinesin family, transporting organelles and moving chromosomes during cell division. The *KIF22* mutation can affect the motor domain of the kinesin, which may lead to the loss of its motor activity. *KIF22* has been confirmed to be highly expressed in multiple cancer tissues (glioma, melanoma, gastric cancer) (49–51), and knocking down *KIF22* can affect the proliferation of cancer cells. However, our research found that in breast cancer, knocking down *KIF22* alone seems to have little effect on the proliferation of cancer cells. We speculate whether it is because of the existence of synthetic lethal pairs that the single mutation has little effect on cancer cells. Through the database, we found that *KRAS* is its synthetic lethal pair. Therefore, we chose to knock down both genes simultaneously and observed significant effects.

*KRAS* is the most prevalent mutated member within the *RAS* family and is recognized as the most common oncogene driver in human malignancies. It stands out as the most well - known oncogene with the highest mutation rate across all cancers (52). *KRAS* is associated with a series of cancers characterized by high lethality, including pancreatic ductal adenocarcinoma (PDAC) (53), non - small cell lung cancer (NSCLC) (54), and colorectal cancer (CRC) (55). It serves as a biomarker indicating poor prognosis for these cancers.

The majority of *KRAS* mutations occur at the G12 and G13 loci of the gene. These mutations have the potential to drive specific pathways that significantly influence tumor biology and can potentially alter the course of tumor development. *KRAS* variants may ultimately prove valuable in classifying tumors into biologically meaningful subgroups. This classification can not only assist in predicting the prognosis but also guide future therapeutic strategies (52).

Research has revealed that patients with *KRAS* mutations and triple - negative breast cancer exhibit luminal progenitor markers. Moreover, the differential expression of angiogenesis and metastasis markers within these markers suggests that tumors harboring *KRAS* variants might represent an aggressive subset of this particular cancer. Previous investigations have shown that the triple - negative breast cancer cell line MDA - MB - 231 carries the *KRAS* G13D mutation, and triple - negative breast cancer (TNBC) is renowned for its high degree of invasiveness and metastatic potential. A substantial body of research exploring the role of *KRAS* in cancer initiation and progression has been continuously refining our understanding of this molecule (56).

Notwithstanding, due to the inherent properties of the *KRAS* protein, targeting *KRAS* has proven to be a formidable challenge in the realm of therapeutics, to the extent that it has been considered “undruggable.” The surface of the *KRAS* protein, with the exception of the *GTP* - binding pocket, lacks other pockets capable of binding small molecules. Targeting the *GTP* - binding pocket is particularly arduous. Under physiological conditions, cells maintain a relatively high concentration of *GTP*, and the affinity between *GTP* and *KRAS* reaches the picomolar level. As a result, *GTP* effectively monopolizes the bottom pocket on the surface of *KRAS*. These innate characteristics render the development of competitive *KRAS* inhibitors extremely difficult, bordering on the realm of the impossible (52).

Consequently, a significant portion of research efforts has been directed towards indirectly targeting *KRAS*. This approach encompasses various strategies, such as targeting its downstream signaling effectors, epigenetic methods (e.g., telomerase inhibitors and RNA interference), and synthetic lethality approaches (e.g., cyclin - dependent kinase inhibitors). Over the past four decades, numerous explorations into indirect targeting of *KRAS* have been carried out. Regrettably, the majority of these strategies have encountered failure, primarily due to their lack of specificity and selectivity, which renders them non - specific and inefficient. Additionally, patients with *KRAS* mutations typically demonstrate a suboptimal response to current standard treatment regimens. Thus, the need to develop effective therapies targeting *KRAS* mutations in *KRAS* - driven cancers remains an urgent and unmet clinical need (57).

In comparison to wild - type *KRAS* cells, tumor cells driven by mutant *KRAS* may exhibit a heightened dependence on certain genes that are essential for maintaining the *KRAS* - driven cell state. One alternative approach involves conducting synthetic lethality screens to target these vulnerable genes. A number of large - scale gene screening studies have been conducted with the aim of identifying genes that are specifically susceptible in *KRAS* - mutated tumors. For example, genes associated with mitotic functions are prominently enriched in the whole - genome RNAi screening of *KRAS* - mutated cells. Additionally, whole - genome gene screening based on the CRISPR/Cas9 system has been extensively employed to identify ideal synthetic lethal targets in Kras - mutated tumors (58).

Regrettably, with the exception of the proteasome system, there is a relatively low degree of overlap among the results of these screening efforts, and these endeavors have not yet translated into successful clinical applications. However, the successful development of *KRAS* (G12C) inhibitors has opened up new avenues of research for synthetic lethality studies related to *KRAS* mutations. The synthetic lethality approach, utilizing whole - genome screening, holds great promise; however, it necessitates more meticulous screening conditions to effectively address the complexity of diverse tumor types. Moreover, effective synthetic lethal targets can be used to define specific subsets of tumors. For example, tumors with *KRAS* mutations, in conjunction with *KEAP1* mutations and the activation of the *NRF2* antioxidant pathway, render them more vulnerable to perturbations in the glutathione pathway (52).

For TNBC patients with *KRAS* mutations (such as G13D in MDA-MB-231 cells), who have difficulty tolerating current *KRAS* (G12C) inhibitors, the *KIF22*-*KRAS* SL strategy provides a supplementary approach. The expressions of *KIF22* and *KRAS* in formalin-fixed paraffin-embedded (FFPE) tissues were detected by conventional immunohistochemistry. These ensure the clinical feasibility of their biomarker tests.

As can be seen from the foregoing, SL activity was essential to the onset, progression, and prognostic evaluation of TNBC. It not only affected the energy metabolism and proliferation ability of cancer cells, but it also had an impact by controlling the immunological microenvironment and metabolic reprogramming to promote tumor invasion and metastasis. By offering new therapies for this highly invasive cancer subtype, focusing on this process gives promise for the treatment of TNBC. Despite current obstacles, SL has the potential to become a crucial part of TNBC treatment as research and technology advance.

## Data Availability

The original contributions presented in the study are included in the article/**Supplementary Material**. Further inquiries can be directed to the corresponding authors.

## Supplementary material

Supplementary Figure 1 Quality control process for single-cell data of TNBC. **(A)** Distribution of nFeature_RNA, nCount_RNA, Percentage.MT, Percentage.HB, and Percentage.Ribosome in TNBC cells. **(B)** Analysis of the correlation between nCount_RNA and nFeature_RNA, nCount_RNA, and percent.MT, percent.HB, per cent.Ribosome. **(C)** Batch-cleaning status of the three TNBC sing-cell datasets.

Supplementary Figure 2 Survival analysis for hallmark genes in METABRIC, GSE58812, and GSE31519.
