# Supplementary figures and images for: Multi-omics comprehensive analysis identified KIF22 and KRAS as highly synthetic lethal pairs for triple-negative breast cancer
Source: Front Oncol. 2026 Feb 6;16:1748954. doi: 10.3389/fonc.2026.1748954 (PMC12920244; doi:10.3389/fonc.2026.1748954)

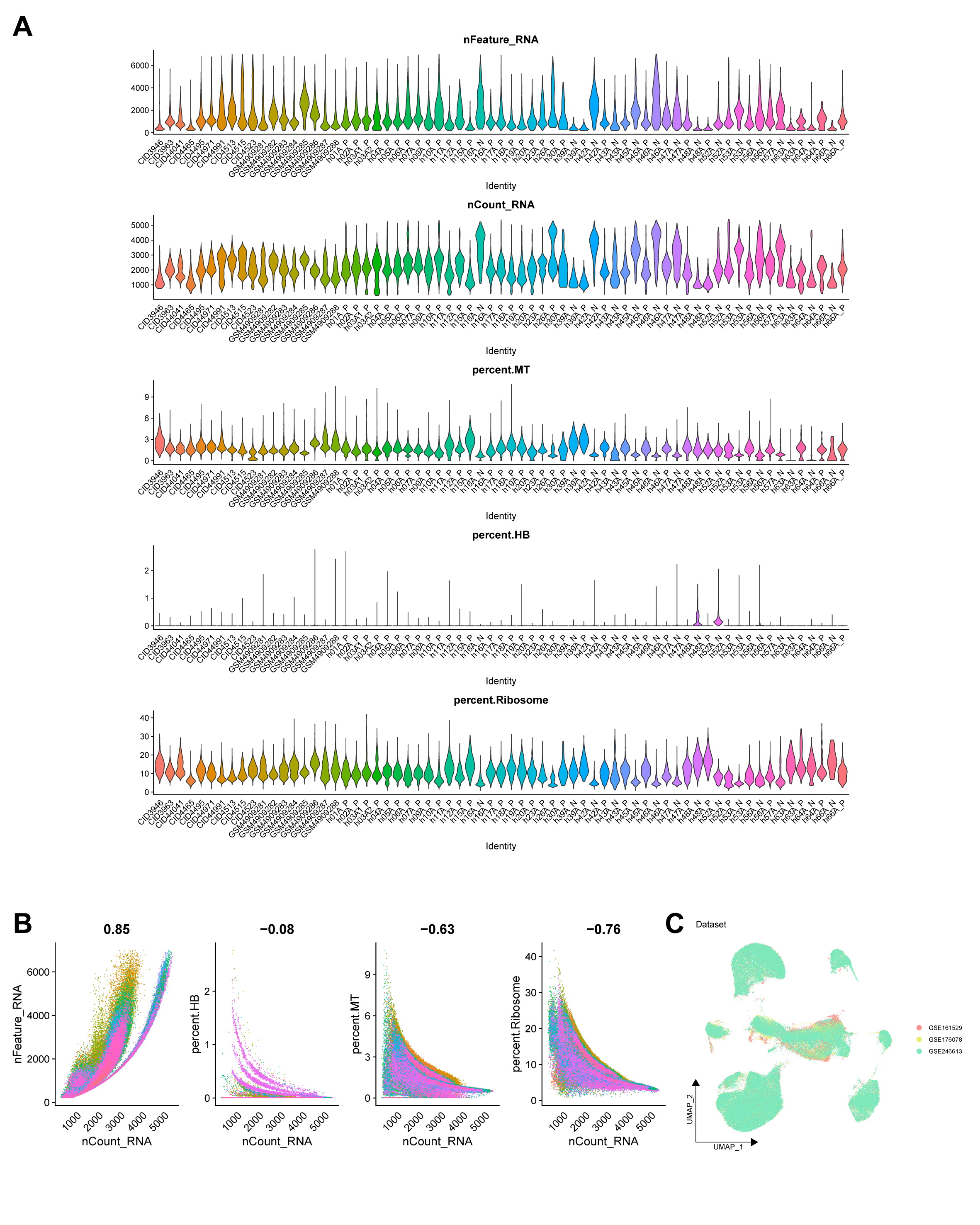

Supplement: Supplementary Figure 1 — Quality control process for single-cell data of TNBC. (A) Distribution of nFeature_RNA, nCount_RNA, Percentage.MT, Percentage.HB, and Percentage.Ribosome in TNBC cells. (B) Analysis of the correlation between nCount_RNA and nFeature_RNA, nCount_RNA, and percent.MT, percent.HB, per cent.Ribosome. (C) Batch-cleaning status of the three TNBC sing-cell datasets. [file Image1.tif]

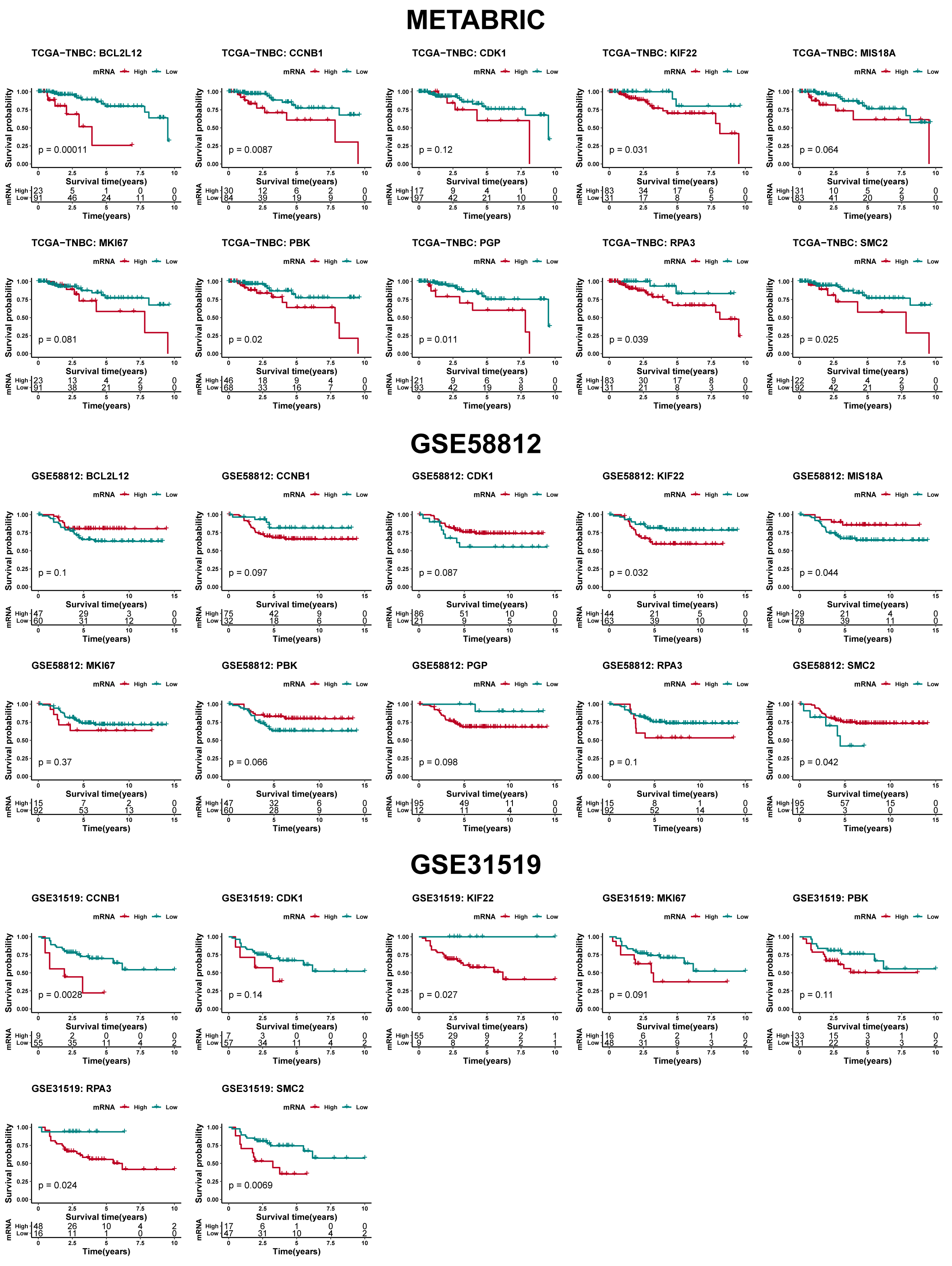

Supplement: Supplementary Figure 2 — Survival analysis for hallmark genes in METABRIC, GSE58812, and GSE31519. [file Image2.tif]
